# Hyperglycemia is associated with duodenal dysbiosis and altered duodenal microenvironment

**DOI:** 10.1038/s41598-023-37720-x

**Published:** 2023-07-07

**Authors:** Aarti Darra, Vandana Singh, Anuraag Jena, Priyanka Popli, Ritambhra Nada, Pankaj Gupta, Sanjay Kumar Bhadada, Anupam Kumar Singh, Vishal Sharma, Anish Bhattacharya, Anurag Agrawal, Usha Dutta

**Affiliations:** 1 CSIR- Institute of Genomics and Integrative Biology, New Delhi, India; 2 Academy of Scientific and Innovative Research (AcSIR), Ghaziabad, 201002 India; 3 Department of Gastroenterology, Post Graduate Institute of Medical Education and Research, Sector-12, Chandigarh, 160012 India; 4 Department of Histopathology, Post Graduate Institute of Medical Education and Research, Chandigarh, India; 5 Department of Radiodiagnosis, Post Graduate Institute of Medical Education and Research, Chandigarh, India; 6 Department of Endocrinology, Post Graduate Institute of Medical Education and Research, Chandigarh, India; 7 Department of Nuclear Medicine, Post Graduate Institute of Medical Education and Research, Chandigarh, India

**Keywords:** Microbiome, Type 2 diabetes

## Abstract

The gut microbiome influences the pathogenesis and course of metabolic disorders such as diabetes. While it is likely that duodenal mucosa associated microbiota contributes to the genesis and progression of increased blood sugar, including the pre-diabetic stage, it is much less studied than stool. We investigated paired stool and duodenal microbiota in subjects with hyperglycemia (HbA1c ≥ 5.7% and fasting plasma glucose > 100 mg/dl) compared to normoglycemic. We found patients with hyperglycemia (n = 33) had higher duodenal bacterial count (*p* = 0.008), increased pathobionts and reduction in beneficial flora compared to normoglycemic (n = 21). The microenvironment of duodenum was assessed by measuring oxygen saturation using T-Stat, serum inflammatory markers and zonulin for gut permeability. We observed that bacterial overload was correlated with increased serum zonulin (*p* = 0.061) and higher TNF-α (*p* = 0.054). Moreover, reduced oxygen saturation (*p* = 0.021) and a systemic proinflammatory state [increased total leukocyte count (*p* = 0.031) and reduced IL-10 (*p* = 0.015)] characterized the duodenum of hyperglycemic. Unlike stool flora, the variability in duodenal bacterial profile was associated with glycemic status and was predicted by bioinformatic analysis to adversely affect nutrient metabolism. Our findings offer new understanding of the compositional changes in the small intestine bacteria by identifying duodenal dysbiosis and altered local metabolism as potentially early events in hyperglycemia.

## Introduction

Type 2 diabetes mellitus (T2DM) is a chronic progressive metabolic disorder characterized by the body’s impaired mechanism of regulating sugar levels. It is rapidly becoming a major health care burden both in developing as well as in developed economies^1^. The disease is preceded by the state of intermediate hyperglycemia known as early diabetes or pre-diabetes. This often goes undiagnosed but presents an opportunity to understand the early effects of disease on biological health of humans. Even though the precise mechanism of disease is not well understood, evidence from experimental and clinical studies show that gut microbiota importantly determine the pathogenesis and course of metabolic diseases including diabetes^2^. Alteration of gut microbiome could induce insulin resistance (IR) in mice, implying an important role of microbiome in the development of diabetes. Similarly, the transplantation of stool microbiota from lean donors to patients with Metabolic syndrome augmented gut bacterial diversity and improved insulin sensitivity (IS)^3^. Such microbiota-attributed impact is at least in part due to altered microbial metabolites, promoting or ameliorating IS. For example, decrease in IS is correlated with decreased abundance of butyrate producing bacteria in the gut^4^.

In fact, host-commensal-pathogen interactions in the gut are also determinants of increased gut permeability, which is an underlying feature of chronic metabolic diseases^5^. It has been shown that impaired gut barrier integrity has a microbial-origin, resulting from a triggered inflammatory response via lipopolysaccharide^6^, which compromises the functionality of tight-junction proteins and causes IR. Besides physical barriers, tissue oxygenation is a less commonly studied factor, such that hypoxia promotes inflammation and insulin resistance.

For a long time, microbiome research has heavily relied on stool samples for studying gut bacterial profile, however stool microbial communities reflect the microbiota resembling the distal gut and do not capture the mucosa associated microbiota (MAM) of the upper gut. Previous investigations have indicated MAM communities in upper gut as risk factors for impaired glucose metabolism^7^. There have only been few studies focused on upper gut, since techniques such as endoscopic duodenal mucosal resurfacing (DMR) and placement of duodenal sleeve, both of which bypass a possibly ‘diseased duodenum’, have shown beneficial improvement in glycemic control^8,9^ and restoration of metabolic homeostasis. While existing evidence around the role of duodenum in diabetes is focused on local nutrient effects via the gut hormonal axis^10^, indirect effects through dysbiosis and immune stimulation are plausible. Recent findings of metformin’s ability to induce glucoregulatory effect via modifying small intestinal bacteria^11^, and mechanism of action of resveratrol acting via duodenal specific Sirt1, enhancing insulin sensitivity^12^ make such research of utmost significance.

The links between hyperglycemia and the factors discussed above are likely to be bidirectional, such that the gut physiology impacts glycemia and glycemic states alter gut physiology. Such relationships, related to MAM, hypoxia, or epithelial permeability, are best explored in the upper gut where the surface area is large, nutrient signaling is coupled to gut-hormonal and gut-neural axes, and host-microbe barriers are not strong as in the colon^13^. Associations starting from pre-diabetes are less likely to suffer from a cause-effect bias where supra-physiological glucose levels of intestinal fluids induce changes in microbiome.

In this study, we investigated the hypothesis that duodenal bacterial dysbiosis and altered microenvironment may be associated with hyperglycemia of pre-diabetes and diabetes (HbA1c ≥ 5.7% and fasting plasma glucose > 100 mg/dl). Prior research examined duodenal bacteria primarily in functional dyspepsia^14^, in cigarette smokers^15^, small intestine bacterial overgrowth^16^, Celiac Disease^17^, pancreatic cancer^18^, and enteropathy^19,20^. This growing body of evidence highlights that upper gut microbial alterations could be causal in disturbing the immune and metabolic signaling contributing to diseases. Our characterization of the overall microbiome in proximal duodenum and paired stool samples in hyperglycemia adds to the lacking body of knowledge about the role of duodenum and the associated microenvironment in early disease state. It is the first such study in Indians. To address the specific question of whether duodenal microbial communities and microenvironment (oxygenation, permeability and systemic inflammation) are different between people with and without hyperglycemia, we recruited subjects who underwent Upper GI endoscopy as part of their standard screening for GI symptoms from PGIMER to obtain duodenum biopsy and paired stool samples for 16S rRNA gene microbiome analysis. Overall, we identified dysbiotic duodenal microbial signatures and provided new insights into the associations of altered microenvironment and microbiota in hyperglycemia.

## Results

### Subject and sample characterstics

A total of 116 samples were sequenced, of which 108 were paired stool and biopsy samples from 54 subjects and remaining 8 were negative controls. Negative controls are samples that contain all DNA extraction required reagents but have no biological material. Paired biopsy and stool samples from each subject were analyzed for 16S microbial analysis. There were 33 hyperglycemic and 21 normoglycemic and none of the subjects had taken any antibiotics in the last 6–8 weeks prior to endoscopy. The endoscopic features of duodenum were found normal in both the study groups. None of the subjects showed severe inflammation or any duodenal related pathology under the histopathological examination. The microbial characterization of a total 108 paired stool and duodenal samples using targeted amplification of V3-V4 of 16SrRNA gene generated 9,926,256 sequences which were assigned to 795 Amplicon Sequence Variants (ASVs).

### Microbial characteristics of duodenum and comparison with stool bacterial profile

**Figure 1:**
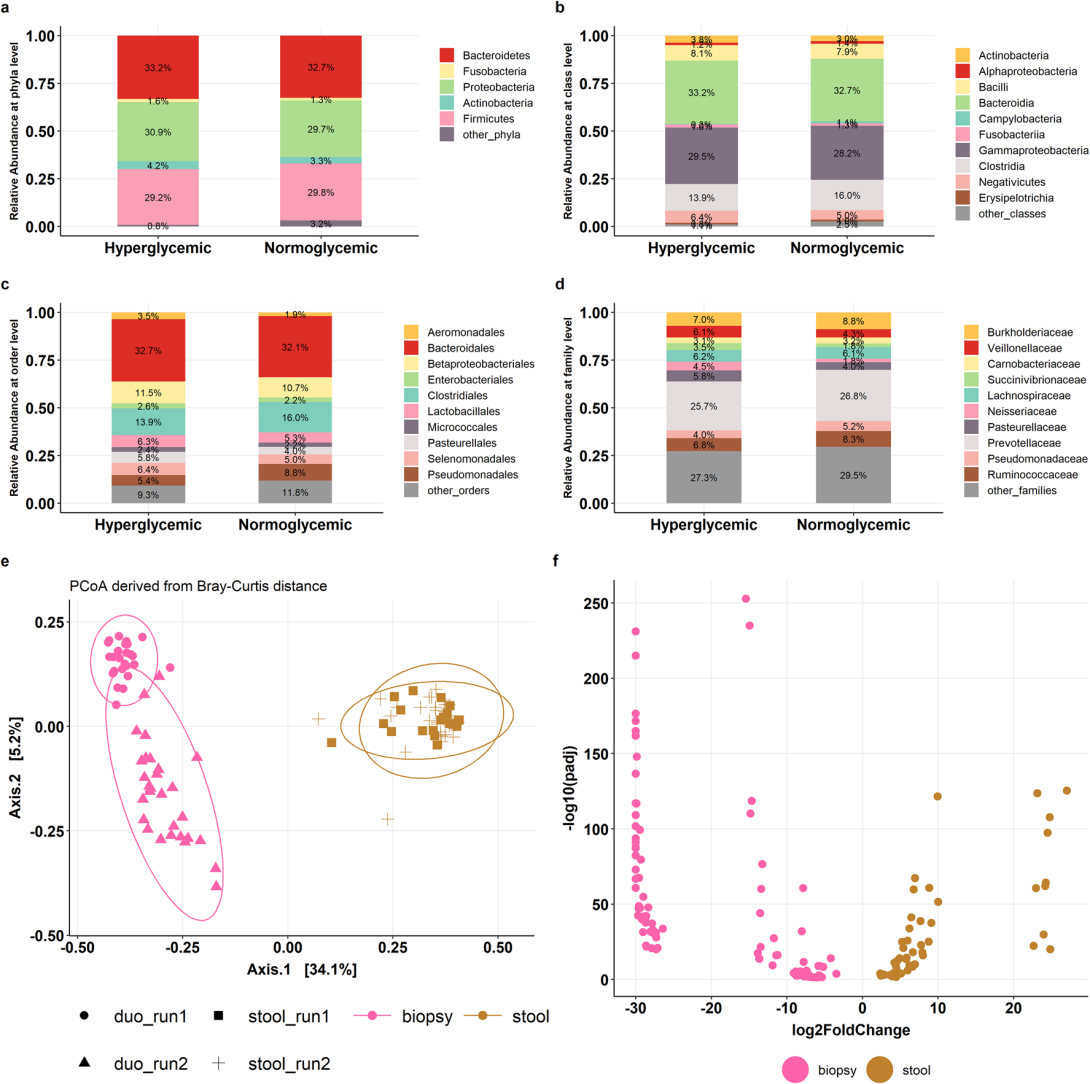
Characteristics of the microbial composition of duodenum and comparison with stool profile. Histogram of taxonomic profiles of the duodenal microbiota between hyperglycemic and normoglycemic subjects at (**a**) phylum, (**b**) class, (**c**) order and (**d**) family level. (**e**) Principal coordinate analysis (PCoA) plot based on Bray–Curtis distance indicating differences in the beta-diversity of stool and duodenum. Proportion of variance is explained by first two principal components and colour of the paired samples indicates the site of the sample, i.e., biopsy (pink) and stool (brown). (**f**) Volcano Plot of estimated log2-fold change in species abundance in duodenal and stool samples (n = 108). p. adj represents multiple testing adjusted p values (FDR). The x-axis represents log2 (foldchange) and y axis represents the statistical significance for each bacterium.

The analysis of 16S amplicon data was carried out to study the bacterial composition of duodenum microbiome in both hyperglycemic and normoglycemic subjects. The relative abundance of dominant phyla was represented by Proteobacteria, Bacteroidetes, Firmicutes, Actinobacteria, and Fusobacteria in both the study groups. There were also minor phyla which included Lentisphaerae, Tenericutes, Epsilonbacteraeota, Chlamydiae, Verrucomicrobia, Patescibacteria, constituting < 1% and < 5% of microbiota in hyperglycemic and normoglycemic subjects respectively (Fig. 1a). A large proportion of reads were assigned to Bacteroidia and Gammaproteobacteria contributing 60% to the duodenal microbiome at class level. Other prominent classes observed were Clostridia, Bacilli, Alphaproteobacteria, Actinobacteria, Campylobacteria, Fusobacteria, Negativicutes, and Erysipelotrichia. Classes with abundances less than 3% abundance were Lentisphaeria, Coriobacteriia, Mollicutes, Chlamydiae, Deltaproteobacteria, Verrucomicrobiae and Saccharimonadia (Fig. 1b). Further, classification at the order level showed 36 taxa in duodenum samples, of which the most abundant were Betaproteobacteriales, Bacteroidales, Pseudomonadales, Lactobacillales, Pasteurellales, Selenomonadales, Micrococcales, Bacillales, Enterobacteriales, and Fusobacteriales (Fig. 1c). Lastly, there were 70 bacterial families in duodenal microbiota and major proportion was comprised of Burkholderiaceae, Prevotellaceae, Pasteurellaceae, Pseudomonadaceae, Neisseriaceae, Veillonellaceae, Carnobacteriaceae, Succinivibrionaceae, Lachnospiraceae, and Streptococcaceae (Fig. 1d).

Next, we investigated whether the microbial composition of duodenum differed from stool. We downsized the phyloseq dataset for varying sequencing depth across samples for principal coordinate analysis (PCoA) and found two distinct clusters representing stool and duodenum (PERMANOVA, R^2^ = 0.381, *p* = 0.001). The PCoA data segregated along Axis 1 and explained 38.1% of the variation in sample clustering (Fig. 1e). The characterization of the microbial differences between stool and duodenal biopsy samples at phylum level demonstrated that the duodenal microbiome had higher relative abundance of Proteobacteria and Firmicutes, constituting nearly 70% of MAM. Compared to the microbial profile in stool, Proteobacteria was approximately fourfold higher (47.9% vs. 13.0%, *p* < 2.2e−16) while Firmicutes was significantly decreased (25.6% vs. 33.4%, *p* = 1.4e-04) in duodenum. The relative percentage of Fusobacteria and Actinobacteria accounted for 8% of MAM and the former remained undetected in stool (3.0% vs. 0.0%, *p* < 2.2e−16) while the latter (6.0% vs. 1.6%, *p* = 1.3e−09) only constituted 1.6% of the stool profile. On the other hand, Bacteroidetes constituted approximately 80% of the stool microbial data with Firmicutes and the proportion of Bacteroidetes was one-third of the stool microbiota (15.9% vs. 50.1%, *p* < 2.2e−16) in duodenum (Supplementary Fig. 1a). Similar significant differences between stool and duodenal bacterial profile were also observed at class, order, and family levels (Supplementary Fig. 1b–d).

We next employed DESeq2 to identify taxonomic differences between stool and biopsy samples at species level (p.adj < 0.05, log2 FoldChange > 2.0) and 153 taxa were found to be differentially abundant in the two sample types (Fig. 1f). There were 97 genera that contributed to the distinctiveness of the duodenal community and the top features were *Delftia sp., Granulicatella sp., Pseudomonas sp., Carnobacterium maltaromaticum, Serratia sp., Granulicatella elegans, Stenotrophomonas sp., Acinetobacter sp., Prevotella nanceiensis,* and *Sphingomonas sp.* (Supplementary Table 1). In contrast, the distinguishing significant taxa in stool were *Megasphaera sp., Bacteroides uniformis, Faecalibacterium prausnitzii, Ruminococcus bromii, Bifidobacterium longum, Dorea longicatena, Bacteroides thetaiotaomicron, Alistipes putredinis, Faecalibacterium sp.,* and *Lactobacillus delbrueckii* (Supplementary Table 2).

### Duodenal mucosal associated microbiome as a predictor of metabolic status

**Figure 2:**
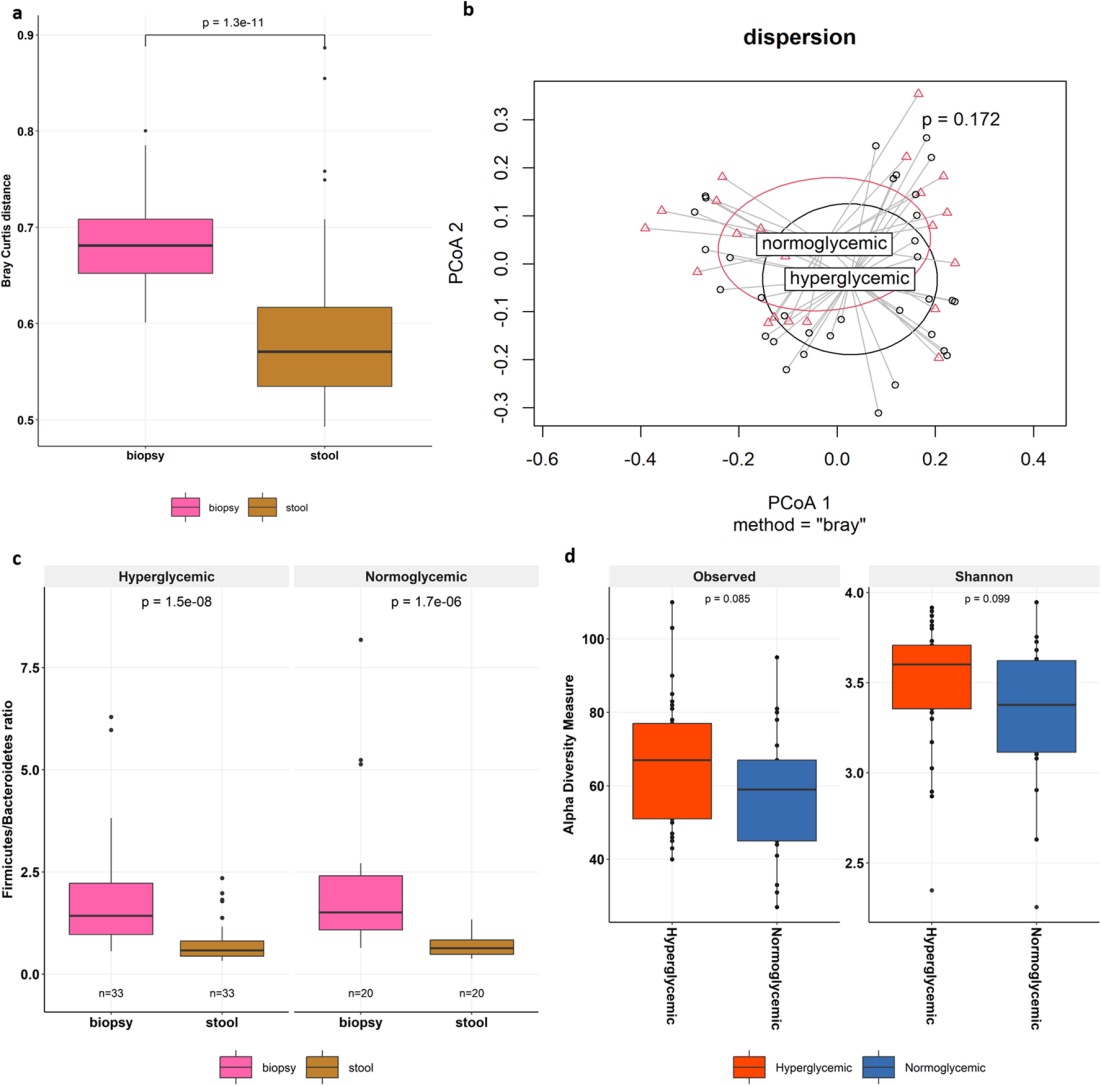
Variation in duodenal microbiome in hyperglycemia (**a**) Box-plot depicting the comparison of within Bray–Curtis distances between stool and duodenum samples and colors of the paired samples indicates the site of the sample, i.e., biopsy (pink) and stool (brown). (**b**) Dispersion plot of PERMANOVA analysis. (**c**) Comparison of Firmicutes/Bacteroidetes ratio between biopsy and stool samples in hyperglycemic and normoglycemic groups.** d**) Boxplot showing the comparison of alpha diversity (Observed richness and Shannon Diversity) between Hyperglycemics and Normoglycemics group in duodenum samples.

We next looked at the microbial heterogeneity between stool and duodenum samples using Bray–Curtis and found that the bacterial composition of duodenum varied more between individuals than that of stool samples (0.68 vs 0.57, *p* = 1.3e−11) (Fig. 2a). Next, we determined whether the observed variance in the beta-diversity was associated with glycemic status or was independent of glycemia. We performed the multivariate analysis (PERMANOVA) and observed a difference in the overall (R^2^ = 2.35, *p* = 0.055) and core (R^2^ = 3.6, *p* = 0.027) bacterial composition of the duodenal samples between hyperglycemic and normoglycemic groups. In contrast, the microbial profile of stool samples was not different between the study groups (overall: R^2^ = 1.98, *p* = 0.318; core: R^2^ = 1.75, *p* = 0.527). To better interpret these findings, we also calculated the within-group dispersion (*p* = 0.172) and found it had no bearing on PERMANOVA results. Thus, bacterial differences in the duodenal samples of hyperglycemic and normoglycemic groups were significantly greater than differences observed within groups (Fig. 2b).

Since the above data suggested biopsy microbiome to be better at distinguishing glycemic status, we next computed the ratio of Firmicutes to Bacteroidetes (F/B), which is an important determinant of altered gut profile. We observed F/B ratio did not differ between the two study groups (Supplementary Fig. 2) but was significantly higher in the duodenal biopsies, when compared to stool of hyperglycemics (1.43 vs. 0.58, p = 1.5e−08) as well as normoglycemics (1.57 vs. 0.63, *p* = 1.7e−06) (Fig. 2c). We also measured the diversity indices and found a marginally higher duodenal bacterial richness (*p* = 0.085) and higher duodenal diversity (*p* = 0.099) in hyperglycemics compared to normoglycemics, however this was not found to be statistically significant (Fig. 2d). On the other hand, no difference in the community richness (*p* = 0.456) or in diversity (*p* = 0.173) was observed in the stool microbiome between the two groups (Supplementary Fig. 3).

### Duodenal bacterial load and bacterial profile in hyperglycemic

**Figure 3:**
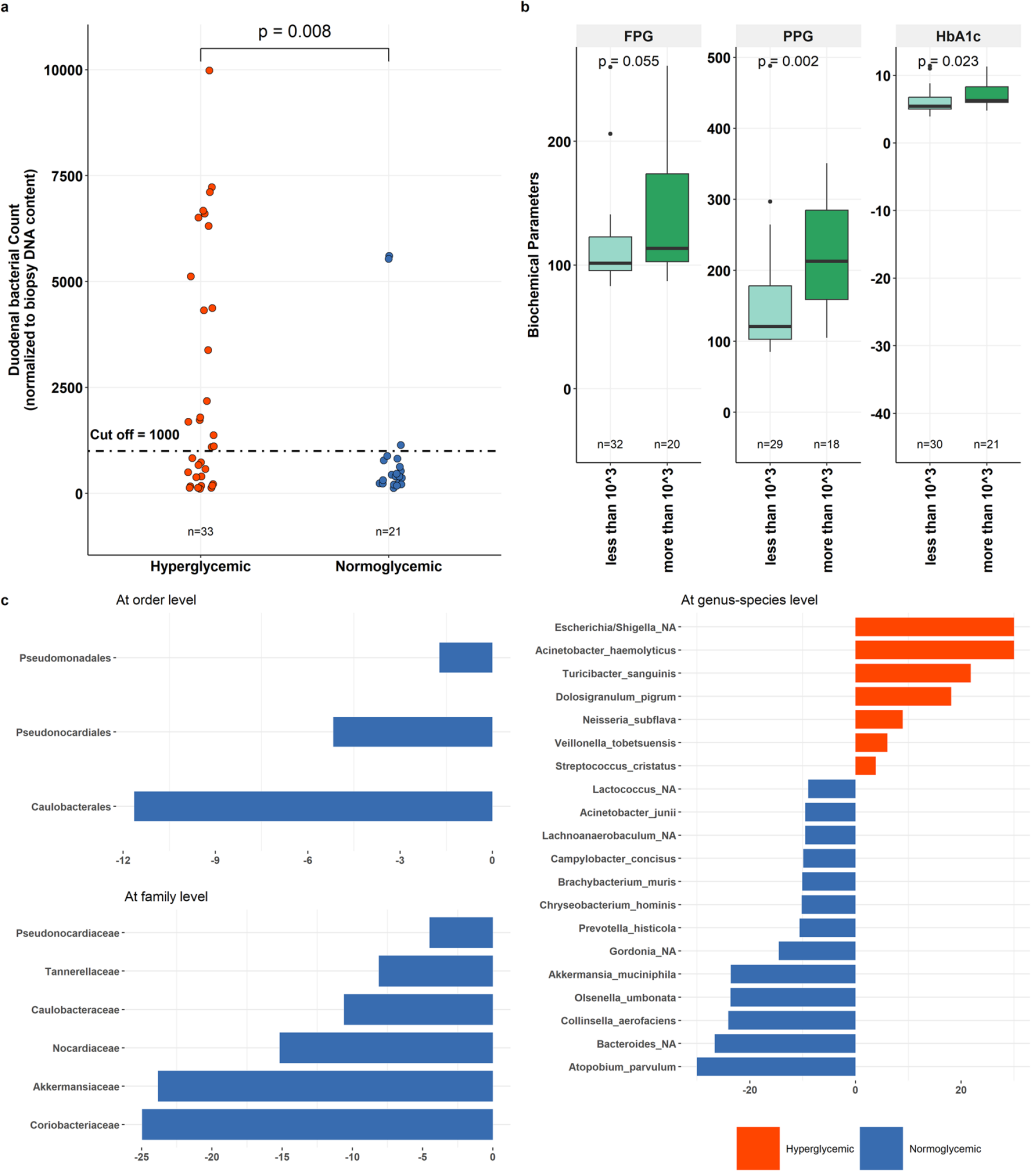
Association of duodenum bacterial load and profile with hyperglycemia (**a**) Dot plot depicting the duodenal bacterial count in hyperglycemic compared to normoglycemic subjects. (**b**) Bar plot displaying the levels of glycemic markers (HbA1c, FPG, PPG) in groups having low and high duodenal bacterial load. (**c**) Histograms showing the differential abundant features in duodenal microbiota obtained using DESeq2 in hyperglycemic and normoglycemic subjects at order, family, and genus-species level.

Based on the above findings and to further explore the microbial alterations quantitatively, we evaluated the total bacterial count in hyperglycemics using q-PCR. Our results showed a significantly higher prevalence of high bacterial load (> 10^3^ bacteria/ng DNA) in the duodenum of hyperglycemics than normoglycemic [18 of 33 (54.5%) vs. 3 of 21 (14.3%), *p* = 0.008] subjects (Fig. 3a).

To validate our findings, we investigated HbA1c, FPG and PPG levels in patients with high and low bacterial load. Substantially, higher HbA1c (6.3% vs. 5.45%, *p* = 0.023), higher FPG (114 mg/dl vs. 102 mg/dl, *p* = 0.055) and higher PPG (213 mg/dl vs. 121 mg/dl, *p* = 0.002) was detected in subjects with high bacterial load (Fig. 3b).

Next, we sought to identify the specific duodenal alterations associated with glycemic status using DESeq2 analysis and identified 20 differentially abundant species. Seven of these species were more prevalent in hyperglycemic while the remaining 13 species were enriched in the duodenum of normoglycemics with varied fold change (FC). Notably, there was 23-fold reduction in the abundance of beneficial bacteria like *Akkermansia muciniphila* (FC = 23.587, p.adj = 1.5E-11) in hyperglycemics. This microbial alteration was exacerbated by the enrichment of pathogenic species such as a 30-fold increase in *Acinetobacter haemolyticus* (FC = 30, p.adj = 8.38E-27) and *Escherichia/Shigella_NA* (FC = 30, p = 2.63E-18) as well as 18-fold rise in *Dolosigranulum pigrum* (FC = 18.138, p.adj = 2.79E-08), and nearly fourfold spike in *Streptococcus cristatus* (FC = 3.830, p.adj = 0.003) in the duodenum of hyperglycemic subjects. At family level, there were 6 families that were enriched in normoglycemic, namely, *Coriobacteriaceae* (FC = 24.962, p.adj = 1.96e-12), *Akkermansiaceae* (FC = 23.842, p.adj = 1.41E-11), *Nocardiaceae* (FC = 15.173, p.adj = 6.58E-05), *Tannerellaceae* (FC = 8.122, p.adj = 0.03E-02), *Caulobacteraceae* (FC = 10.602, p.adj = 0.002) and *Pseudonocardiaceae* (FC = 4.511, p.adj = 0.038). Furthermore, orders such as *Pseudomonadales* (FC = 1.721, p.adj = 0.001), *Pseudonocardiales* (FC = 5.170, p.adj = 0.014) and *Caulobacterales* (FC = 11.640, p.adj = 0.001) were elevated in normoglycemic. No differences were found at phylum or class level in the duodenal microbiome of hyperglycemic and normoglycemic subjects (Fig. 3c).

### Reduced oxygenation and altered immune state in hyperglycemia

**Figure 4:**
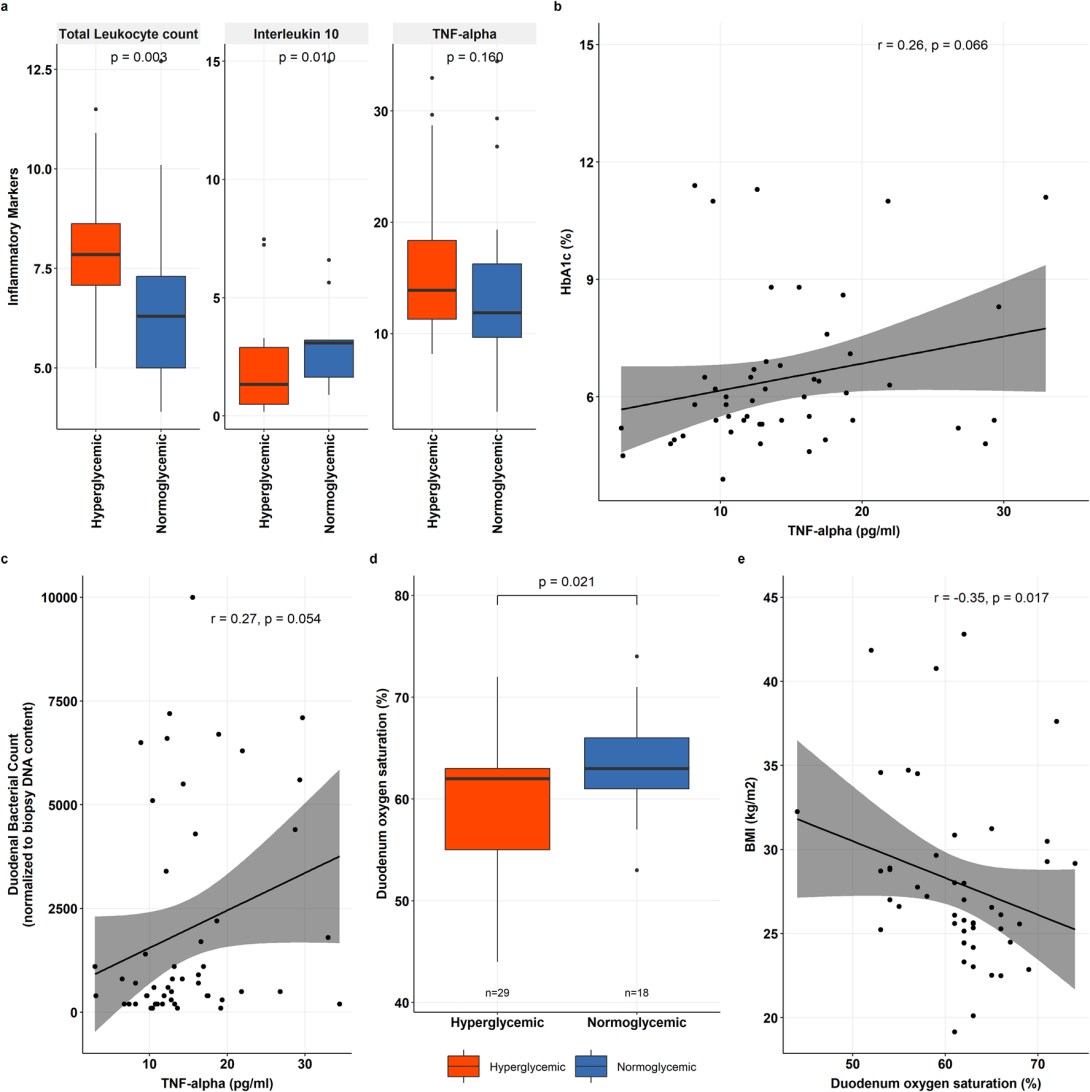
Microenvironment alterations in hyperglycemia (**a**) Boxplot showing the comparison of inflammatory markers (TNF-α, IL-10, TLC) between hyperglycemics and normoglycemics group. (**b**) Graph displaying the spearman correlation between levels of serum TNF-α and HbA1c. (**c**) Graph displaying the spearman correlation between levels of serum TNF-α and Duodenal bacterial count. (**d**) Boxplot comparing the duodenal mucosal oxygen saturation in hyperglycemics and normoglycemics group. (**e**) Graph displaying the spearman correlation between Duodenal oxygen saturation and BMI.

In response to the enrichment of pathogenic organisms observed in the duodenal profile of hyperglycemia, we assessed the inflammatory status of these subjects. We found hyperglycemics to have higher total leukocyte count (TLC) than normoglycemic subjects (*p* = 0.031). In addition, the levels of the inflammation resolution cytokine, Interleukin 10 (IL-10) were found to be significantly reduced (*p* = 0.015) (Fig. 4a). There was a marginal elevation in the level of inflammatory cytokine, Tumor Necrosis Factor-α (TNF-α) (13.9 pg/ml versus 11.9 pg/ml, *p* = 0.162) in hyperglycemics, which showed a trend of positive correlation with HbA1c (r_s_ = 0.26, *p* = 0.066) (Fig. 4b). Additionally, we identified the relation between the inflammatory state and the increased duodenal bacterial load, and we discovered that TNF-α showed a positive link with the duodenal bacterial levels (r_s_ = 0.27, *p* = 0.054) (Fig. 4c).

Further, to understand the relationship of inflammation and duodenal bacterial burden contributing to the microenvironment changes, we quantified duodenal mucosal oxygenation and permeability. The tissue oxygenation was assessed using T-Stat and demonstrated a significantly lower mucosal oxygen saturation in the duodenum of hyperglycemic subjects (59.8% vs. 63.7%, *p* = 0.021) compared to normoglycemic (Fig. 4d). Interestingly, the duodenal oxygenation was also found to be negatively associated with BMI (r_s_ = −0.35, *p* = 0.017) (Fig. 4e).

Next, to determine whether the small intestinal permeability was associated with glycemia, we measured the levels of serum zonulin^21,22^. We observed subjects with normoglycemia and those with hyperglycemia did not differ in terms of zonulin levels (41.5 ng/ml vs 40.3 ng/ml, *p* = 0.830) (Supplementary Fig. 4). However, higher zonulin levels showed a positive trend towards increased duodenal bacterial load (r_s_ = 0.36, *p* = 0.061) and zonulin correlations also extended to obesity associated parameters such as body mass index (r_s_ = 0.65, *p* = 0.01E−02), waist circumference (r_s_ = 0.47, *p* = 0.009), and hip circumference (r_s_ = 0.53, *p* = 0.003) (Supplementary Table 3).

### Association of glycemic markers with the duodenal metabolic pathways

**Figure 5:**
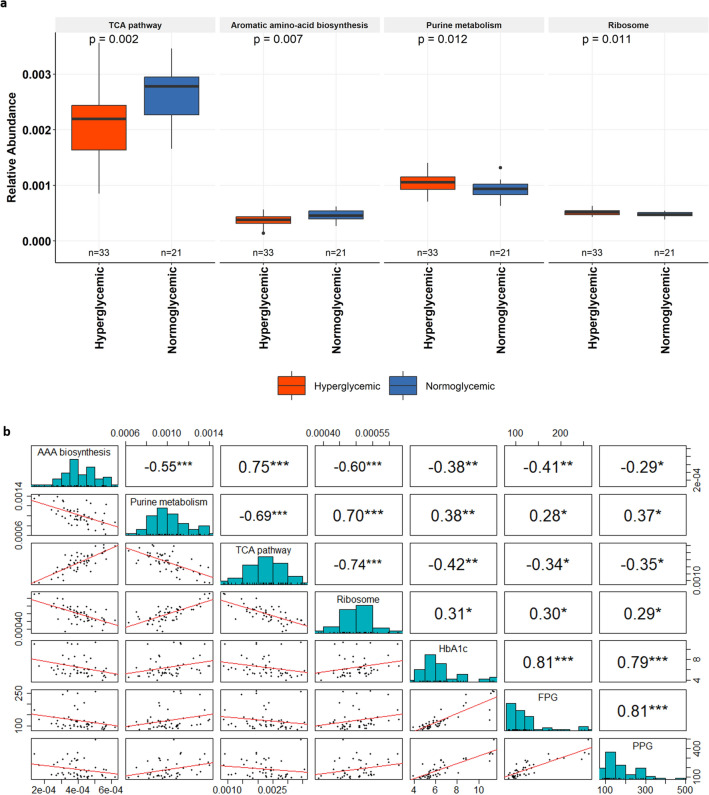
Correlation between glycemic markers and altered metabolic pathways predicted using duodenal 16S data. (**a**) Box-plot showing the comparison of important metabolic pathways between hyperglycemic and normoglycemic. The x-axis displays the glycemic status of samples and y-axis shows the relative abundance of metabolic pathways. (**b**) Spearman’s rank correlation matrix revealing the significant correlations between the glycemic markers (FPG: Fasting plasma glucose, PPG: Postprandial glucose and HbA1c: Glycated hemoglobin) and predicted metabolic pathways (TCA pathway, AAA (Aromatic amino acid synthesis) pathway, Purine metabolism and Ribosome). The upper panel indicates statistically significant correlation coefficients represented with stars (*p* ≤ 0.05). The diagonal depicting the distribution of the variables and the lower panel shows the scatter graphs with detailed illustration of the relation between the variables.

To gain a deeper insight into the roles played by the duodenal microbiome, we employed the obtained 16S duodenal bacterial profile data of hyperglycemics and normoglycemics to predict the metabolic pathways using Tax4Fun. There were eleven metabolic KEGG pathways that were differentially abundant between the two groups (Supplementary Fig. 5). We found that the pathways of purine/pyrimidine metabolism and translation, that control cellular energy needs and nucleic acid repair, were elevated in hyperglycemic groups. However, abundance of pathways belonging to aromatic amino acid biosynthesis (phenylalanine, tyrosine, and tryptophan), and of TCA cycle pathway were found to be significantly reduced in hyperglycemia (Fig. 5a).

Next, we examined the relationship between the predicted duodenal metabolic pathways and the glycemic markers using the spearman correlation analysis (Supplementary Fig. 6). Particularly, the TCA cycle pathway showed a negative association with the glycemic markers; HbA1c (r_s_ = −0.42, *p* < 0.05), FPG (r_s_ = −0.34, *p* < 0.05), and PPG (r_s_ = −0.35, *p* < 0.05). Similarly, another important pathway, involved in the synthesis of aromatic amino acids exhibited an unfavourable relationship with HbA1c (r_s_ = −0.38, *p* < 0.05), FPG (r_s_ = −0.41, *p* < 0.05), and PPG (r_s_ = −0.29, *p* < 0.05). Besides these negative associations, a favourable link of purine metabolism with HbA1c (r_s_ = 0.38, *p* < 0.05), FPG (r_s_ = 0.28, *p* < 0.05), and PPG (r_s_ = 0.37, *p* < 0.05) was found by association analysis, which also extended to pathway related to translation, HbA1c (r_s_ = 0.31, *p* < 0.05), FPG (r_s_ = 0.30, *p* < 0.05), and PPG (r_s_ = 0.29, *p* < 0.05) (Fig. 5b).

## Discussion

Over the last two decades, easy accessibility and high bacterial mass have made stool to be the sample of interest in gut microbiome studies. As the field is evolving, limitations of using stool as a surrogate for the entire gut have become clear. Stool fails to capture the less abundant yet metabolically active microbes, residing in the upper small bowel. These microbes are involved in nutrient absorption system, and are very relevant to understanding metabolic health risks. In the developing world, where modifiable adverse environmental factors such as sanitation and hygiene may play bigger roles than genetic risks, it is important to explicitly study upper gut flora and potential dysbiosis. Yet, there has been no systematic study of the upper gut microbiome in the Indian population. In this cross-sectional study, we examined duodenal MAM and the microenvironment & investigated their relationship with hyperglycemia. We also provided analysis of the differences between paired duodenal and stool microbiome.

As expected, there was a clear separation between the microbial profiles of stool and duodenum as seen previously^23^. The duodenal biopsy had major phyla as Proteobacteria, even in normoglycemic group^24^ and Bacteroidetes with Firmicutes constitute a dominant fraction of stool microbiota. While firmicutes form a common link, the composition of Firmicutes in duodenum was different from that in stool and was mainly characterized by families *Carnobacteriaceae, Bacilliceae*, and *Acidaminococcaceae* (Supplementary Fig. 7). The genera-level classification of duodenum samples was represented by *Delftia sp., Granulicatella sp., Pseudomonas sp., Carnobacterium maltaromaticum, Serratia sp., Granulicatella elegans, Stenotrophomonas sp., Acinetobacter sp., Prevotella nanceiensis and Sphingomonas sp.* whereas stool samples had abundance of *Megasphaera sp., Bacteroides uniformis, Faecalibacterium prausnitzii, Ruminococcus bromii, Bifidobacterium longum, Dorea longicatena, Bacteroides thetaiotaomicron, Alistipes putredinis, Faecalibacterium sp.,* and *Lactobacillus delbrueckii*. The data suggested that stool samples did not provide any approximation of the proximal upper gut microbiota. Many of our findings are consistent with the results of Li *et al*^25^, where duodenal luminal and mucosal bacteria were analysed and mucosal diversity was shown to be greater with a unique microbial signature.

We found that duodenal microbiota may be clinically more relevant in understanding host metabolic health than stool microbiota. Despite the small significant difference of 1.3% in the HbA1c levels between hyperglycemic and normoglycemic subjects, we found that the duodenal mucosal bacterial profile showed discriminatory signal while no differences were detected in the stool. In duodenum, families *Coriobacteriaceae* and *Akkermansiaceae* were found significantly more abundant in normoglycemic compared to hyperglycemic. These bacterial families have a role in the transformation of bile acid^26^ and maintaining the acid pool^27^ respectively, both of which are critical part of a microbially driven mechanism of regulating glucose metabolism. Furthermore, disproportion in the abundance of beneficial micro-organisms is linked with diabetes. The opportunistic pathogens such as *Acinetobacter haemolyticus*^28^, *Dolosigranulum pigrum*^29^, *Escherichia/Shigella sp.*^30^ and *Streptococcus cristatus* were significantly more prevalent in the duodenum of hyperglycemics compared to normoglycemics. On the contrary, previous report by Pellegrini et al.^31^ found that T1DM had a unique immunological and duodenal microbiological profile, and reported decreased prevalence of pathogenic micro-organisms. This may indicate that the pathophysiology of T1DM and T2DM are different.

This is further substantiated by the significant enrichment of genera that were associated with gut and metabolic improvement in normoglycemic, such as *Lactococcus*, which is shown to reduce several potential opportunistic microbes^32^, *Akkermansia muciniphilia,* which confers anti-inflammatory benefits and preserves the mucosal architecture as well as integrity^33,34^. Another genus is *Atopobium*, a Hydrogen-sulphide (H_2_S) producer^35^, that contributes to small intestinal motility^36^, which is an important determinant of blood glucose^37^.

The stool profile was consistent with the already available data of an Indian gut^38^. Families *Prevotellaceae, Succinivibrionceae, Lachospiraceae, Ruminococcaceae* dominate in both normoglycemic and hyperglycemic. At class level, Bacteroidia, and Clostridia were prominent and to a lesser extent, Gammaproteobacteria, Actinobacteria and Negativicutes classes were also present. The stool microbial community was similar between the groups while the duodenal profile showed important microbial differences associated with glycemia. This could be caused by the duodenum and pancreas shared embryological, functional, vascular and structural relationship, which could affect the entero-insular signaling, crucial for maintaining glucose homeostasis^39^.

We found greater inter-individual variability in the mucosal microbial composition similar to the findings of Vaga et al.^40^. Moreover, the F/B ratio, which is one of the hallmarks of the dysbiotic gut profile^41^ remained unchanged between hyperglycemic and normoglycemic but was found to be higher in duodenum, indicating that disease specific cues were detected at a higher signal strength in the duodenum than in stool.

To better understand the full potential of upper gut microbes to modulate human health, the factors influencing the interactions between mucosal microbes and their environment are worth exploring. Indeed, the prominent one is the amount of duodenal mucosal bacteria due to the aggregates of lymphoid follicles residing underneath^42^. Excess of bacteria may be related to immune activation that may have an etiological role in diabetes^43^. The apparent high duodenal bacterial load observed in hyperglycemia and the correlation between bacterial burden and inflammatory marker (r_s_ = 0.054, *p* < 0.05) supports the above observation. Further, the positive correlation between TNF-α and HbA1c corroborated such an association. We also noted reduced anti-inflammatory IL-10 levels, which is shown to increase insulin sensitivity and affect peripheral glucose metabolism^44,45^. Together, our results suggested the duodenal microenvironment to be inflammatory, overpopulated with bacteria, and with reduced anti-inflammatory signals in hyperglycemic condition.

Another important microenvironment parameter influenced by intestinal bacteria is gut permeability. In this study, we measured serum zonulin, a marker of small intestinal permeability. Research by Asmar et al.^46^, described the zonulin pathway as a mechanism of innate defense, flushing out the microbes with water using hydrostatic pressure gradients. In our data, we did not find any difference in zonulin levels between the groups, as observed in previous reports^47^ or any association with taxa. Interestingly, higher zonulin levels showed a trend of association with increased mucosal bacterial load. The relatively small size of the dataset for zonulin measurements is likely to underestimate the relationship between permeability and microbial markers. To our observation, our data was at least sufficient to provide a robust validation to the previously addressed associations between zonulin and anthropometric measurements related to obesity^48^.

Besides physical barrier, tissue oxygenation maintains the functional balance of mucosa and directly shapes microbial community as exemplified by the increased amount of dissolved oxygen after RYGB surgery that favors the proliferation of Proteobacteria phyla^49^. On the other hand, microbes also actively participate in shaping the mucosal environment by degrading mucus or triggering AMP production^50^. Our observations showed a lower duodenal tissue oxygenation in hyperglycemia. This decrease could be due to the reduced perfusion of the gut or due to increased local utilization of oxygen^51^, which could not be distinguished by the data. Thus, the question of whether decreased oxygenation is a cause, or consequence, of the altered microbial profile or metabolism in the duodenum, remains to be explored. It is interesting that the oxygen saturation values for both groups are lower than those reported in Western studies^52^. Since this is the first such study from South Asia, it is conceivable that bacterial overload and lower oxygenation may be frequent in low-middle income tropical countries. Future multi-centric studies should explore this further.

There is not a considerable body of clinical data to link the pathogenesis of metabolic diseases to microbial disturbances in the upper gut or the associated environment. In this study, we have investigated this relationship and predicted the metabolic state of the duodenum in hyperglycemic condition. In duodenum, the main source of energy is amino acids such that 30 to 50 percent of amino acids do not enter portal circulation^53^. To our observation, we found diminished ability of synthesizing aromatic amino acids (phenylalanine, tyrosine, and tryptophan) in hyperglycemia which could impact host physiology. Small molecules with potent biological effects are derived from these aromatic amino acids. For example, tryptophan derived metabolites (viz. Tryptamine, indole 3-acetic acid) are important modulators of epithelial integrity and mucosal immune response^54^. Another key pathway implicated in altered β-cell metabolism is the TCA cycle pathway^55^, which was found to be reduced in the duodenum of hyperglycemic. Such abberant change in local duodenal metabolism under high glycemic condition could be brought on by altered microbial metabolism. The changed metabolic state could possibly also disrupt the core imbued duodenal pathways of nutrition sensing and entero-insular signalling, critical for maintaining glucose homeostasis^56^.

We acknowledge some important limitations of the study. First, the age of the normoglycemic group was significantly lower than that of the hyperglycemics. This could be attributed to the increased prevalence of hyperglycemia with aging. To account for effect of age, we have adjusted the age variable while analyzing data using DESeq2, as previously reported in small bowel microbiome study^57^. Secondly, the study was performed in a limited number of subjects and directionality of associations could not be concluded from the data. Third, zonulin levels represent the small intestinal permeability and are not specific only to duodenum but in the jejunum as well, thus it cannot be directly linked to duodenal microbiome. Fourth, the study is cross-sectional in nature and captures the intestinal bacterial composition at a single time point.

There are multiple factors which define strengths of the study. First, we included microenvironment variables (oxygenation, permeability, inflammation) alongside the microbiome, unlike most prior duodenal microbiome studies. Second, the use of negative controls during DNA extraction provided an additional confidence to the results of the study, as low biomass biopsy samples are prone to contamination. Third, we analyzed hyperglycemia without remaining restricted only to diabetics, to study the early disease associated microbial changes which could be leading to diabetes and most importantly, the paired assessment of duodenal and stool bacterial profile was performed for the enrolled patients.

In conclusion, variability in the duodenal mucosa associated microbiota profile showed association with glycemia. The assessment of small intestinal mucosa associated microbiota, with the inclusion of a quantitative approach, merits deeper investigation as a health determinant. Understanding the compositional changes in the small intestine bacterial population is an important area of research, especially in the context of human nutrition and to find future link to human health and disease.

## Materials and methods

### Study population

In this cross-sectional study, we recruited 69 subjects after obtaining informed consent in 2018–19 according to the guidelines of the Declaration of Helsinki, 1975. The study was conducted in compliance with the Indian Council for Medical Research guidelines, Good Practices for Clinical Research in India. The study was approved by the Ethics Committee of PGIMER (IEC No: PGI/IEC/2018/000237, dated: 12/03/2018). Written informed consent was obtained from all study participants.

The inclusion criteria included adult subjects (18–64 years) who underwent Upper GI endoscopy as part of their standard screening for GI symptoms, anemia as well as to rule out celiac disease or GI bleeding. Subjects who were pregnant, lactating, had celiac disease, colon cancer and gastrointestinal (GI) bleeding were excluded. After eliminating subjects with a history of consumption of antibiotics in the last 6–8 weeks, absence of paired duodenum/stool sample and low read sequencing depth (< 2000), 54 subjects with paired samples were finally analyzed. Based on the blood parameters; fasting plasma glucose (FPG; mg/dl), postprandial glucose (PPG; mg/dl) and glycated hemoglobin (HbA1c, %), subjects with FPG > 100 mg/dl and HbA1c ≥ 5.7%; were classified as hyperglycemic, while those with FPG ≤ 100 mg/dl and HbA1c < 5.7%; were classified as normoglycemic^58^. Overall, 33 established hyperglycemic and 21 normoglycemic were included in the study. As shown in Table 1, there were statistically significant differences in age and clinical traits of the groups, with unvarying BMI.

**Table 1:** Demographic and clinical characteristics of study subjects, grouped by gender within glycemic group.

Groups	Hyperglycemic			Normoglycemic			Between groups
Variables	Male (n = 19)	Female (n = 14)	*p* value	Male (n = 17)	Female (n = 4)	*p* value	*p* value
Demography							
Age (years, SD)	46.8 (8.37)	49.1 (8.24)	0.476	38.9 (12.6)	41.5 (5.80)	0.346	0.004
Body mass index (kg/m^2^) (median, IQR)	25.6 [24.3, 30.1]	27.9 [26.7, 33.2]	0.152	25.6 [22.9, 26.1]	29.1 [28.9, 30.6]	0.006	0.142
Clinical characteristics							
Glycated hemoglobin (%) (median, IQR)	6.60 [5.95, 7.98]	6.50 [6.20, 8.80]	0.378	5.05 [4.80, 5.40]	5.35 [5.28, 5.43]	0.127	< 0.001
Fasting plasma glucose (mg/dl) (median, IQR)	130 [118, 165]	117 [103, 127]	0.254	96.3 [92.7, 104]	90.8 [87.2, 94.2]	0.203	< 0.001
Post prandial glucose (mg/dl) (Median, IQR)	222 [169, 281]	199 [133, 275]	0.592	106 [96.1, 115]	95.8 [90.5, 108]	0.659	< 0.001

### DNA extraction and sequencing of clinical samples

Duodenal biopsies from the D2 region were obtained endoscopically using the Olympus endoscope GIF-HQ190. The biopsy was collected in RNAlater for microbial analysis and for histology in 10% formalin. The stool sample was collected in a polypropylene container and stored at − 80 °C without preservatives. Blood samples were collected in additive free red capped vacutainers to obtain sera. The extraction of DNA from stool and biopsy samples was performed using Qiagen Stool Mini kit and DNeasy Blood and Tissue kit respectively, with the additional step of bead-beating^59^. Negative controls were maintained in each batch of DNA extraction. For library preparation, V3-V4 region of 16S rRNA was selected and paired-end sequencing was performed as per the Illumina approved protocol for 16 s rRNA marker gene survey^60^ on MiSeq platform (V3, 600 cycles, Illumina). The final library was reconstituted using the Tris buffer at 4 nM. The loading concentration of the library was 8 pM with 15–20% PhiX.

### Sequence analysis

Using DADA2 pipeline^61^, raw sequences were filtered with following parameters; maxEE = c(2,3), trunclen = c(275,220), trimleft = c(17,21), maxN = 0, truncQ = 2, rm.phix = T and the settings for remaining steps were default. The amplicon sequence variant (ASV) tables from each of the two duodenum and stool runs were then combined and taxonomy was assigned using SILVA database (Release 132). Subsequently, the phyloseq object containing the phylogenetic tree, constructed using the Neighbor joining method, was decontaminated using the "decontam" package after chimera removal. In decontam, there are two approaches for decontamination; i.e., frequency and prevalence-based. The combined approach was applied for stool, and the recommended prevalence-based method was used for low-biomass duodenum samples. Taxa that appeared more than three times across samples and had a prevalence of more than 5% were included for total microbiome analysis, and taxa that had a prevalence of at least 50% was used for core microbial analysis.

### Calculations and statistical analysis

R was used for statistical analysis and ggplot2, ggpubr packages were used for data visualization. Based on the distribution, clinical parameters were expressed as medians with interquartile range [Q1-Q3] or mean (standard deviation). Histogram was constructed to represent the top 10 taxa with the highest proportion at different taxonomic levels in duodenum and stool samples. At a rarefied depth of 13,188 ASVs/sample, alpha diversity measures (Observed Species and Shannon Diversity) were calculated. To compare paired quantitative samples, non-parametric Wilcoxon Sign Rank test was applied. For beta diversity, the rarefied object was hellinger transformed and Principal Coordinate Analysis (PCoA) was used to discover grouping patterns. Variability in the community structure was assessed by permutational analysis of variance (PERMANOVA) using adonis function in vegan package^62^. The inter-sample Bray Curtis measurements were computed using the microbiome package. The relative abundance at phylum level was used to calculate Firmicutes/Bacteroidetes ratio in stool and biopsy samples.

Using the DESeq2 package, the differential abundance analysis on untransformed species level phyloseq object was performed between stool and biopsy samples. The differential abundance analysis was also performed between hyperglycemic and normoglycemic to find characteristic features at different taxonomic levels with respect to glycemia in stool and duodenum samples. Age and sequencing batch effects were adjusted wherever required and p < 0.05 was regarded statistically significant.

### Quantification of bacterial load

A series of tenfold dilutions of recombinant plasmid (full length 16S rRNA gene of E. coli cloned in pRS315 vector) were used to create a standard curve. The universal 16S primers, eubF (GTGSTGCAYGGYTGTCGTCA) and eubR (ACGTCRTCCMCACCTTCCTC)^63^ were used with 15 ng of template in a Roche LightCycler 480 to set up a reaction. The amplification conditions were 3 min at 95 °C followed by 40 cycles of 95 °C for 15 s and data collection at 60 °C for 20 s. Using a previously recommended cut-off of normalized 10^3^ bacteria for determination of small intestinal bacterial overgrowth^64^, the bacterial count data of hyperglycemic and normoglycemic was categorized as ‘high bacterial load’ and ‘low bacterial load’ and compared using Chi-square test with Yates correction. Further, the levels of HbA1c, FPG and PPG were compared between the 'high bacterial load’ and ‘low bacterial load’ groups.

### Measurement of inflammatory markers

The Luminex assay was performed using MILLIPLEX MAP Human Cytokine/Chemokine HCYTOMAG-60 K-8 Panel to measure the concentrations of Interferon-γ, Interleukin-1β, Tumor Necrosis Factor-α, Interleukin-8, Interleukin-4, Interleukin-5, and Interleukin-10. The software xPONENT 3.1 (Luminex corp.) was used to examine the data and the levels of inflammatory markers were compared between the two study groups. The relationship between these factors and anthropometric measurements, glycemic markers, and bacterial load was examined by Spearman correlation test.

### Measurement of gut perfusion and permeability

The FDA-approved T-Stat (Spectros), was used to measure duodenal mucosal oxygen saturation (D2). Only 47 patient’s mucosal oxygenation could be measured due to technical difficulties (29 hyperglycemic; 18 normoglycemic). After suppressing peristalsis with a single dosage of hyoscine butylbromide (Buscopan- 10 mg/ml), multiple measurements were taken and an average of three readings per individual was used for analysis. The gut permeability assay using serum zonulin was performed using ELISA kit (Immundiagnostik AG, Bensheim, Germany)^65^. Only pre-endoscopic sera samples were used to measure zonulin (16 hyperglycemic and 12 normoglycemic). The spearman correlation test was used to test association between zonulin and anthropometric measurements and bacterial load.

### Prediction of metabolic pathways

Based on the 16S sequencing data, Tax4Fun (t4f.) in "themetagenomics" package was used to predict metabolic capabilities of duodenal bacteria. We mapped the taxonomic abundance and annotated the functional content using KEGG orthologues (https://www.genome.jp/kegg/ko.html). The samples were normalized by overall functional abundance (to compare the relative abundance of predicted pathways between samples) and the anticipated functional profile was adjusted for 16S copy number variability. A linear regression model adjusted for sequencing batch effects and fluctuation in the fraction of unused taxonomic units (no hit in the reference database), was used to find pathways that were linked to glycemic status. Subset of data was plotted as correlation graphs using psych package and the entire data was represented as heatmap using the pheatmap package.

## Supplementary Information

Supplementary Information.

## Data Availability

Raw sequences and associated metadata have been deposited on NCBI public repository [Bio project- PRJNA793189, Study ID: SRP352925].

## Abbreviations

T2DM: Type-2 diabetes mellitus
MAM: Mucosa associated microbiota
DMR: Duodenal mucosal resurfacing
ASVs: Amplicon sequence variants
IL-10: Interleukin 10
TNF-α: Tumor necrosis factor-α
TLC: Total leukocyte count
F/B ratio: Firmicutes/bacteroidetes ratio
FPG: Fasting plasma glucose
PPG: Postprandial plasma glucose
HbA1c: Glycated hemoglobin
GI: Gastrointestinal
IS: Insulin sensitivity

## References

[1] Abdul M, Khan B, Hashim MJ, King JK, Govender RD, Mustafa H (2020). Epidemiology of type 2 diabetes—global burden of disease and forecasted trends. J. Epidemiol. Global Health..

[2] Zhou W, Sailani MR, Contrepois K, Zhou Y, Ahadi S, Leopold SR (2019). Longitudinal multi-omics of host–microbe dynamics in prediabetes. Nature.

[3] Vrieze A, Van Nood E, Holleman F, Salojärvi J, Kootte RS, Bartelsman JFWM (2012). Transfer of intestinal microbiota from lean donors increases insulin sensitivity in individuals with metabolic syndrome. Gastroenterology..

[4] van Deuren T, Blaak EE, Canfora EE (2022). Butyrate to combat obesity and obesity-associated metabolic disorders: Current status and future implications for therapeutic use. Obes. Rev..

[5] Fasano A (2020). All disease begins in the (leaky) gut: role of zonulin-mediated gut permeability in the pathogenesis of some chronic inflammatory diseases. F1000Research.

[6] Cani PD, Amar J, Iglesias MA, Poggi M, Knauf C, Bastelica D (2007). Metabolic endotoxemia initiates obesity and insulin resistance. Diabetes.

[7] Demmer RT, Trinh P, Rosenbaum M, Li G, LeDuc C, Leibel R (2019). Subgingival microbiota and longitudinal glucose change: The oral infections, glucose intolerance and insulin resistance study (ORIGINS). J Dent Res..

[8] Van Baar ACG, Holleman F, Crenier L, Haidry R, Magee C, Hopkins D (2020). Endoscopic duodenal mucosal resurfacing for the treatment of type 2 diabetes mellitus: One-year results from the first international, open-label, prospective, multicentre study. Gut.

[9] Mingrone G, Van Baar ACG, Devière J, Hopkins D, Moura E, Cercato C (2021). Safety and efficacy of hydrothermal duodenal mucosal resurfacing in patients with type 2 diabetes: The randomised, double-blind, sham-controlled, multicentre REVITA-2 feasibility trial. Gut.

[10] Theodorakis MJ, Carlson O, Michopoulos S, Doyle ME, Juhaszova M, Petraki K (2006). Human duodenal enteroendocrine cells: Source of both incretin peptides, GLP-1 and GIP. Am. J. Physiol. Endocrinol. Metab..

[11] Bauer PV, Duca FA, Waise TMZ, Rasmussen BA, Abraham MA, Dranse HJ (2018). Metformin alters upper small intestinal microbiota that impact a glucose-SGLT1-sensing glucoregulatory pathway. Cell Metab..

[12] Côté CD, Rasmussen BA, Duca FA, Zadeh-Tahmasebi M, Baur JA, Daljeet M (2015). Resveratrol activates duodenal Sirt1 to reverse insulin resistance in rats through a neuronal network. Nat. Med..

[13] Donaldson GP, Lee SM, Mazmanian SK (2015). Gut biogeography of the bacterial microbiota. Nat. Rev. Microbiol..

[14] Jung H, Talley NJ (2018). Role of the duodenum in the pathogenesis of functional dyspepsia: A paradigm shift. J. Neurogastroenterol. Motil..

[15] Shanahan ER, Shah A, Koloski N, Walker MM, Talley NJ, Morrison M (2018). Influence of cigarette smoking on the human duodenal mucosa-associated microbiota. Microbiome..

[16] Shin AS, Gao X, Bohm M, Lin H, Gupta A, Nelson DE (2019). Characterization of proximal small intestinal microbiota in patients with suspected small intestinal bacterial overgrowth: A cross-sectional study. Clin. Transl. Gastroenterol..

[17] Cheng J, Kalliomäki M, Heilig HG, Palva A, Lähteenoja H, de Vos WM (2013). Duodenal microbiota composition and mucosal homeostasis in pediatric celiac disease. BMC Gastroenterol..

[18] Kohi S, Macgregor-Das A, Dbouk M, Yoshida T, Chuidian M, Abe T (2020). Alterations in the duodenal fluid microbiome of patients with pancreatic cancer. Clin. Gastroenterol. Hepatol..

[19] Shulzhenko N, Dong X, Vyshenska D, Greer RL, Gurung M, Vasquez-Perez S (2018). CVID enteropathy is characterized by exceeding low mucosal IgA levels and interferon-driven inflammation possibly related to the presence of a pathobiont. Clin. Immunol..

[20] Chen RY, Kung VL, Das S, Hossain MS, Hibberd MC, Guruge J (2020). Duodenal microbiota in stunted undernourished children with enteropathy. N. Engl. J. Med..

[21] Mörkl S, Lackner S, Meinitzer A, Mangge H, Lehofer M, Halwachs B (2018). Gut microbiota, dietary intakes and intestinal permeability reflected by serum zonulin in women. Eur. J. Nutr..

[22] Kaczmarczyk M, Löber U, Adamek K, Węgrzyn D, Skonieczna-Żydecka K, Malinowski D (2021). The gut microbiota is associated with the small intestinal paracellular permeability and the development of the immune system in healthy children during the first two years of life. J. Transl. Med..

[23] Leite GG, Weitsman S, Parodi G, Celly S, Sedighi R, Sanchez M, Morales W, Villanueva-Millan MJ, Barlow GM, Mathur R, Lo SK (2020). Mapping the segmental microbiomes in the human small bowel in comparison with stool: A REIMAGINE Study. Digest. Dis. Sci..

[24] Nardelli C, Granata I, D’Argenio V, Tramontano S, Compare D, Guarracino MR (2020). Characterization of the duodenal mucosal microbiome in obese adult subjects by 16S rRNA sequencing. Microorganisms.

[25] Li G, Yang M, Zhou K, Zhang L, Tian L, Lv S (2015). Diversity of duodenal and rectal microbiota in biopsy tissues and luminal contents in healthy volunteers. J. Microbiol. Biotechnol..

[26] Zhao X, Zhang Z, Hu B, Huang W, Yuan C, Zou L (2018). Response of gut microbiota to metabolite changes induced by endurance exercise. Front. Microbiol..

[27] Juárez-Fernández M, Porras D, Petrov P, Román-Sagüillo S, García-Mediavilla MV, Soluyanova P (2021). The synbiotic combination of *Akkermansia muciniphila* and quercetin ameliorates early obesity and NAFLD through gut microbiota reshaping and bile acid metabolism modulation. Antioxidants..

[28] Bai L, Zhang S, Deng Y, Song C, Kang G, Dong Y (2020). Comparative genomics analysis of *Acinetobacter haemolyticus* isolates from sputum samples of respiratory patients. Genomics.

[29] Lécuyer H, Audibert J, Bobigny A, Eckert C, Jannière-Nartey C, Buu-Hoï A (2007). *Dolosigranulum pigrum* causing nosocomial pneumonia and Septicemia. J. Clin. Microbiol..

[30] Tetz G, Brown SM, Hao Y, Tetz V (2019). Type 1 Diabetes: an association between autoimmunity, the dynamics of gut amyloid-producing *E. coli* and their phages. Sci Rep..

[31] Pellegrini S, Sordi V, Bolla AM, Saita D, Ferrarese R, Canducci F (2017). Duodenal mucosa of patients with type 1 diabetes shows distinctive inflammatory profile and microbiota. J. Clin. Endocrinol. Metab..

[32] Carvalho R, Vaz A, Pereira FL, Dorella F, Aguiar E, Chatel J-M (2018). Gut microbiome modulation during treatment of mucositis with the dairy bacterium Lactococcus lactis and recombinant strain secreting human antimicrobial PAP. Sci. Rep..

[33] Depommier C, Everard A, Druart C, Plovier H, Van Hul M, Vieira-Silva S (2019). Supplementation with *Akkermansia muciniphila* in overweight and obese human volunteers: A proof-of-concept exploratory study. Nat. Med..

[34] Bian X, Wu W, Yang L, Lv L, Wang Q, Li Y (2019). Administration of *Akkermansia muciniphila* ameliorates dextran sulfate sodium-induced ulcerative colitis in mice. Front. Microbiol..

[35] Mottawea W, Chiang C-K, Mühlbauer M, Starr AE, Butcher J, Abujamel T (2016). Altered intestinal microbiota–host mitochondria crosstalk in new onset Crohn’s disease. Nat. Commun..

[36] Linden DR (2014). Hydrogen sulfide signaling in the gastrointestinal tract. Antioxid. Redox Signal..

[37] Chaikomin R, Rayner CK, Jones KL, Horowitz M (2006). Upper gastrointestinal function and glycemic control in diabetes mellitus. World J. Gastroenterol..

[38] Kulkarni AS, Kumbhare SV, Dhiraj DP, Shouche YS (2019). Mining the core gut microbiome from a sample indian population. Indian J. Microbiol..

[39] Del Castillo E, Meier R, Chung M, Koestler DC, Chen T, Paster BJ (2019). The microbiomes of pancreatic and duodenum tissue overlap and are highly subject specific but differ between pancreatic cancer and noncancer subjects. Cancer Epidemiol. Biomark. Prev..

[40] Vaga S, Lee S, Ji B, Andreasson A, Talley NJ, Agréus L (2020). Compositional and functional differences of the mucosal microbiota along the intestine of healthy individuals. Sci. Rep..

[41] Magne F, Gotteland M, Gauthier L, Zazueta A, Pesoa S, Navarrete P (2020). The firmicutes/bacteroidetes ratio: A relevant marker of gut dysbiosis in obese patients?. Nutrients.

[42] Wells JM, Rossi O, Meijerink M, van Baarlen P (2011). Epithelial crosstalk at the microbiota—Mucosal interface. Proc. Natl. Acad. Sci. U.S.A..

[43] Yan L, Mu B, Pan D, Shi Y, Yuan J, Guan Y (2020). Association between small intestinal bacterial overgrowth and beta-cell function of type 2 diabetes. J. Int. Med. Res..

[44] Hong E-G, Ko HJ, Cho Y-R, Kim H-J, Ma Z, Yu TY (2009). Interleukin-10 prevents diet-induced insulin resistance by attenuating macrophage and cytokine response in skeletal muscle. Diabetes.

[45] Straczkowski M, Kowalska I, Nikolajuk A, Krukowska A, Gorska M (2005). Plasma interleukin-10 concentration is positively related to insulin sensitivity in young healthy individuals. Diabetes Care.

[46] Asmar RE, Panigrahi P, Bamford P, Berti I, Not T, Coppa GV (2002). Host-dependent zonulin secretion causes the impairment of the small intestine barrier function after bacterial exposure. Gastroenterology.

[47] Thaiss CA, Levy M, Grosheva I, Zheng D, Soffer E, Blacher E (2018). Hyperglycemia drives intestinal barrier dysfunction and risk for enteric infection. Science.

[48] Ohlsson B, Orho-Melander M, Nilsson P (2017). Higher levels of serum zonulin may rather be associated with increased risk of obesity and hyperlipidemia, than with gastrointestinal symptoms or disease manifestations. Int. J. Mol. Sci..

[49] Tremaroli V, Karlsson F, Werling M, Ståhlman M, Kovatcheva-Datchary P, Olbers T (2015). Roux-en-Y gastric bypass and vertical banded gastroplasty induce long-term changes on the human gut microbiome contributing to fat mass regulation. Cell Metab..

[50] De Weirdt R, Van de Wiele T (2015). Micromanagement in the gut: microenvironmental factors govern colon mucosal biofilm structure and functionality. npj Biofilms Microbiomes.

[51] Friedman ES, Bittinger K, Esipova TV, Hou L, Chau L, Jiang J (2018). Microbes vs chemistry in the origin of the anaerobic gut lumen. Proc. Natl. Acad. Sci. U.S.A..

[52] Benaron DA, Parachikov IH, Friedland S, Soetikno R, Brock-Utne J, van der Starre A (2004). Continuous, noninvasive, and localized microvascular tissue oximetry using visible light spectroscopy. Anesthesiology..

[53] Wu G, Knabe DA, Flynn NE (2005). Amino acid metabolism in the small intestine: biochemical bases and nutritional significance. Biology of Growing Animals.

[54] Liu Y, Hou Y, Wang G, Zheng X, Hao H (2020). Gut microbial metabolites of aromatic amino acids as signals in host-microbe interplay. Trends Endocrinol. Metab..

[55] Haythorne E, Rohm M, van de Bunt M, Brereton MF, Tarasov AI, Blacker TS (2019). Diabetes causes marked inhibition of mitochondrial metabolism in pancreatic β-cells. Nat. Commun..

[56] Duca FA, Bauer PV, Hamr SC, Lam TKT (2015). Glucoregulatory relevance of small intestinal nutrient sensing in physiology, bariatric surgery, and pharmacology. Cell Metab..

[57] Leite G, Pimentel M, Barlow GM, Chang C, Hosseini A, Wang J (2021). Age and the aging process significantly alter the small bowel microbiome. Cell Rep..

[58] Kumar S, Anand A, Nagarathna R, Kaur N, Sivapuram MS, Pannu V (2021). Prevalence of prediabetes, and diabetes in Chandigarh and Panchkula region based on glycated haemoglobin and Indian diabetes risk score. Endocrinol. Diab. Metab..

[59] Moen AEF, Tannæs TM, Vatn S, Ricanek P, Vatn MH, Jahnsen J (2016). Simultaneous purification of DNA and RNA from microbiota in a single colonic mucosal biopsy. BMC Res. Notes.

[60] Illumina. 16S Metagenomic Sequencing Library. Illumina.com. 2013;1–28.

[61] Callahan BJ, McMurdie PJ, Rosen MJ, Han AW, Johnson AJA, Holmes SP (2016). DADA2: High-resolution sample inference from Illumina amplicon data. Nat. Methods.

[62] Anderson MJ (2017). Permutational Multivariate Analysis of Variance (PERMANOVA). Wiley StatsRef: Statistics reference online.

[63] Maeda H, Fujimoto C, Haruki Y, Maeda T, Kokeguchi S, Petelin M (2003). Quantitative real-time PCR using TaqMan and SYBR Green for *Actinobacillus actinomycetemcomitans*, *Porphyromonas gingivalis*, *Prevotella intermedia*, *tetQ* gene and total bacteria. FEMS Immunol. Med. Microbiol..

[64] Leite G, Morales W, Weitsman S, Celly S, Parodi G, Mathur R (2020). The duodenal microbiome is altered in small intestinal bacterial overgrowth. PLOS ONE..

[65] Aasbrenn M, Lydersen S, Farup PG (2020). Changes in serum zonulin in individuals with morbid obesity after weight-loss interventions: A prospective cohort study. BMC Endocr Disord..

