# Supplementary material for: Hyperglycemia is associated with duodenal dysbiosis and altered duodenal microenvironment
Source: Sci Rep. 2023 Jul 7;13:11038. doi: 10.1038/s41598-023-37720-x (PMC10329043; doi:10.1038/s41598-023-37720-x)
Supplement: Supplementary file 1 — Supplementary Information. [file 41598_2023_37720_MOESM1_ESM.pdf]

Supplementary Information

Hyperglycemia is associated with duodenal dysbiosis and altered duodenal microenvironment

Authors:

Aarti Darra, MSc[+, #], Vandana Singh, MSc[+, #], Anuraag Jena, MD, DM[^,1], Priyanka Popli, MSc[^,1], Ritambhra Nada, MD[^,2], Pankaj Gupta, MD[^,3], Sanjay Kumar Bhadada, MD, DM[^,4], Anupam Kumar Singh, MD, DM[^,1], Vishal Sharma, MD, DM[^,1], Anish Bhattacharya, DNB, PhD[^,5], Anurag Agrawal, MD, PhD[+, #], Usha Dutta, MD, DM[^, 1]

Supplementary Figures

a)

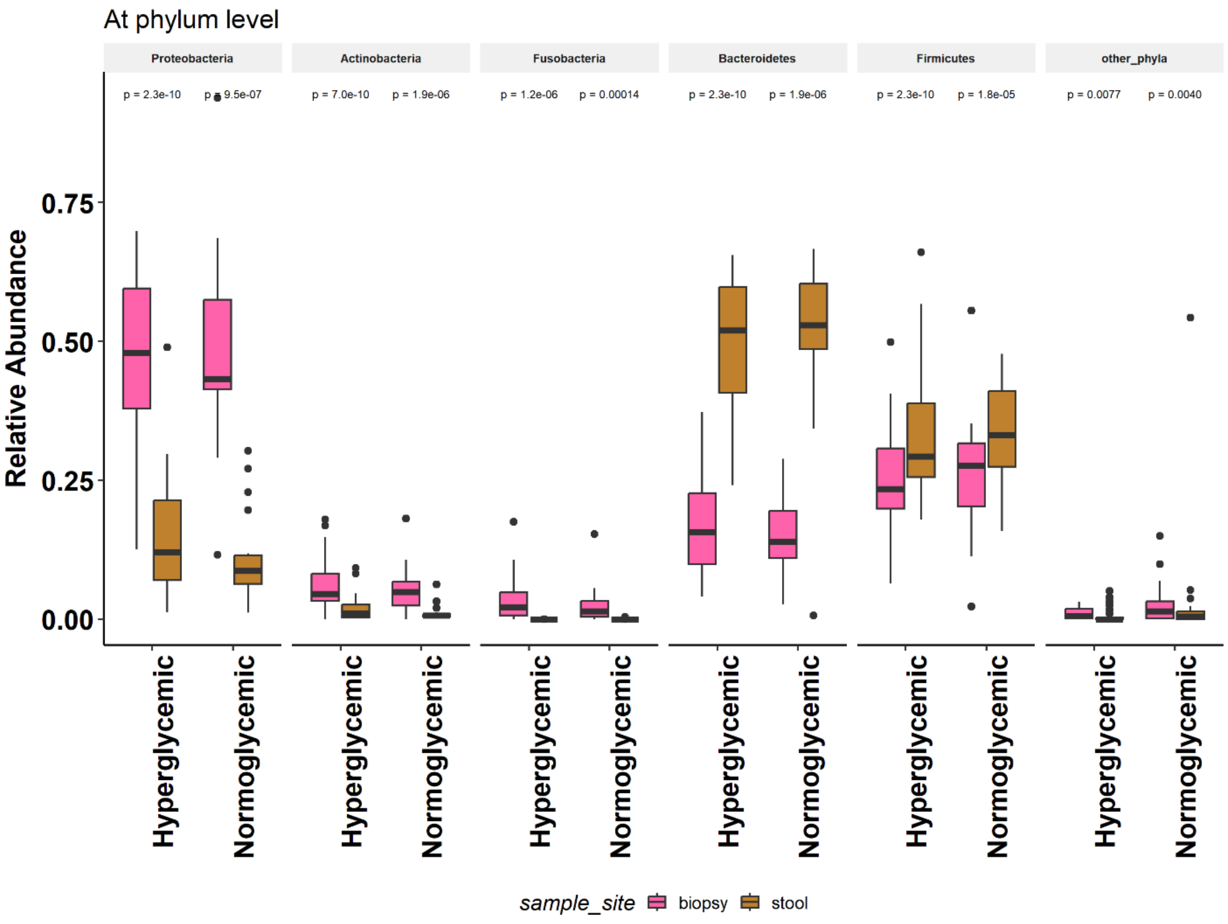

b)

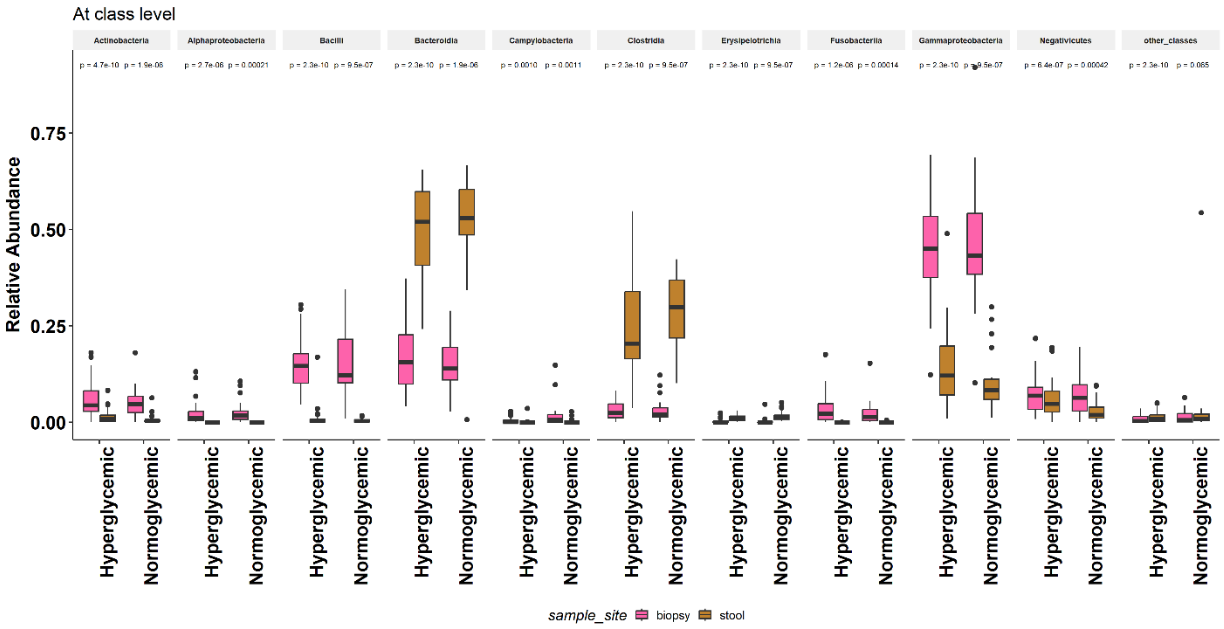
At class level
Actinobacteria
p = 4.7e-10 p = 1.9e-06
Alphaproteobacteria
p = 2.7e-06 p = 0.00021
Bacilli
p = 2.3e-10 p = 9.5e-07
Bacteroidia
p = 2.3e-10 p = 1.9e-06
Campylobacteria
p = 0.0010 p = 0.0011
Clostridia
p = 2.3e-10 p = 9.5e-07
Erysipelotrichia
p = 2.3e-10 p = 9.5e-07
Fusobacteriia
p = 1.2e-06 p = 0.00014
Gammaproteobacteria
p = 2.3e-10 p = 9.5e-07
Negativicutes
p = 6.4e-07 p = 0.00042
other_classes
p = 2.3e-10 p = 0.065
Relative Abundance
0.75
0.50
0.25
0.00
Hyperglycemic
Normoglycemic
sample_site
biopsy
stool

c)

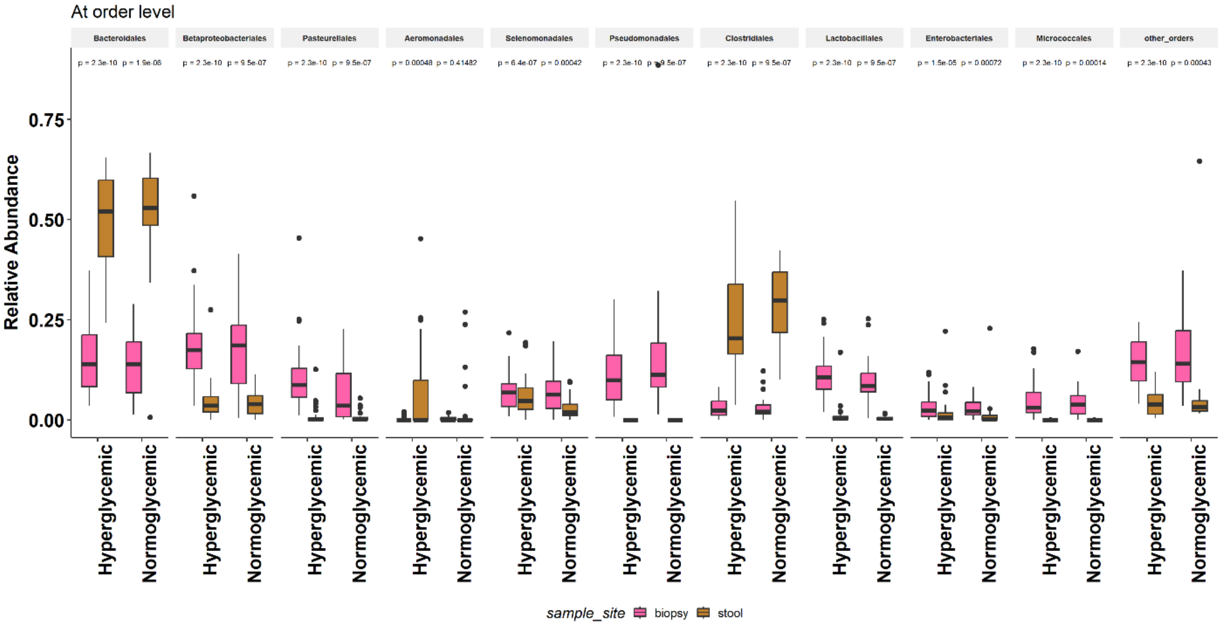
At order level
Bacteroidales
p = 2.3e-10 p = 1.9e-06
Betaproteobacteriales
p = 2.3e-10 p = 9.5e-07
Pasteurellales
p = 2.3e-10 p = 9.5e-07
Aeromonadales
p = 0.00048 p = 0.41482
Selenomonadales
p = 6.4e-07 p = 0.00042
Pseudomonadales
p = 2.3e-10 p = 9.5e-07
Clostridiales
p = 2.3e-10 p = 9.5e-07
Lactobacillales
p = 2.3e-10 p = 9.5e-07
Enterobacteriales
p = 1.5e-05 p = 0.00072
Micrococcales
p = 2.3e-10 p = 0.00014
other_orders
p = 2.3e-10 p = 0.00043
Relative Abundance
0.75
0.50
0.25
0.00
Hyperglycemic
Normoglycemic
sample_site
biopsy
stool

d)

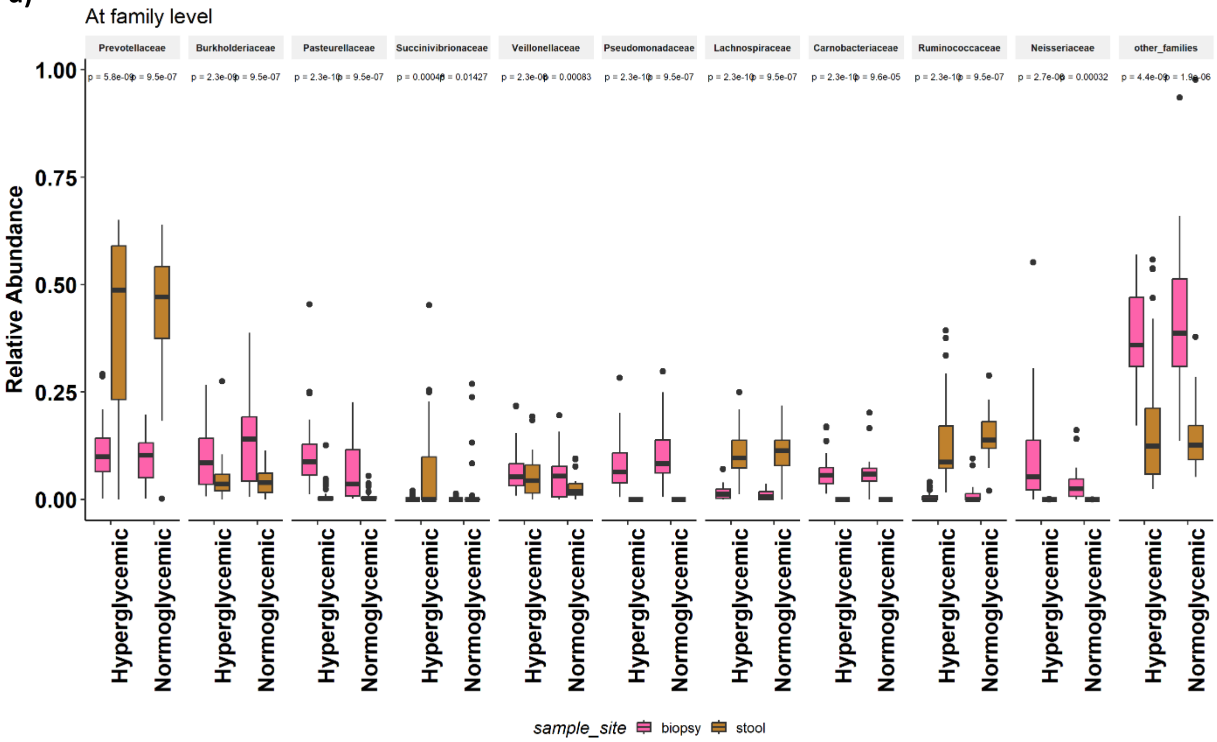

<u>Supplementary Figure 1</u>: Boxplot showing the comparison of top taxa between hyperglycemics and normoglycemics group in stool and duodenum samples at **a)** Phylum **b)** Class **c)** Order and **d)** Family level.

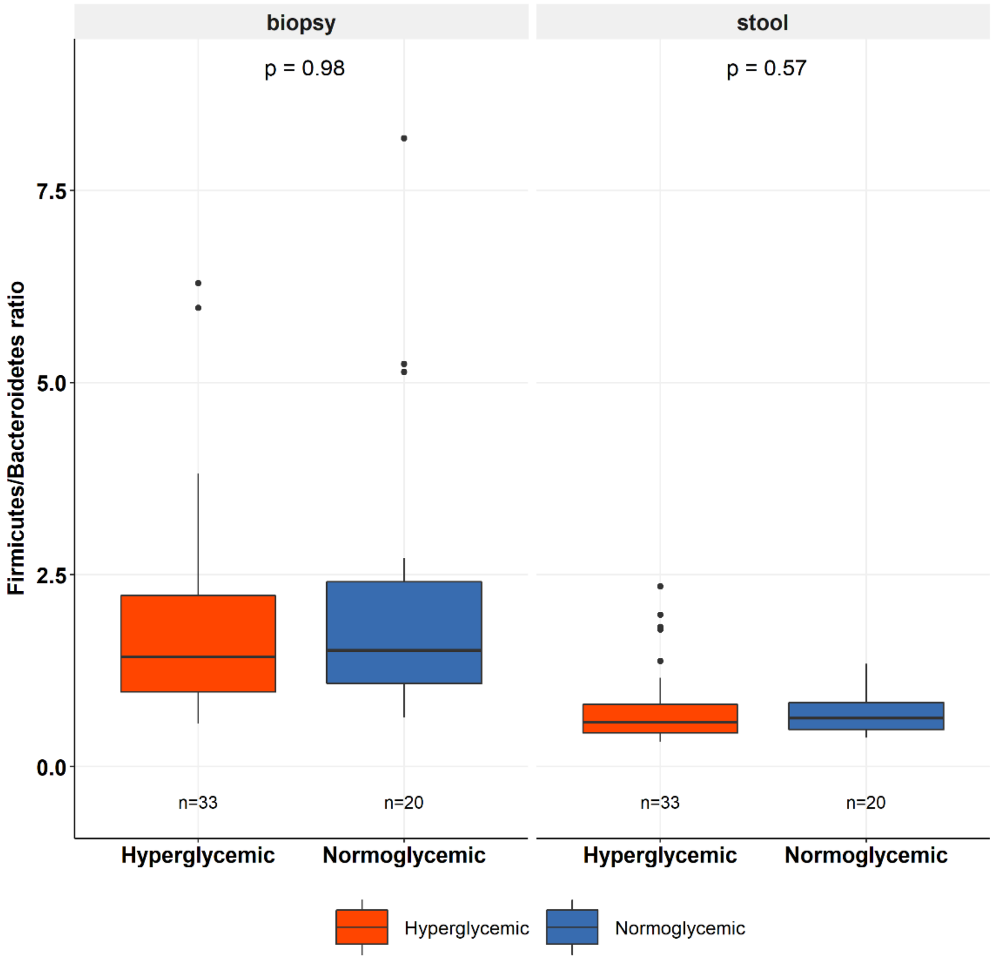

<u>Supplementary Figure 2</u>: Boxplot showing the comparison of Firmicutes to Bacteroidetes ratio between hyperglycemics and normoglycemics group, segregated by sample site.

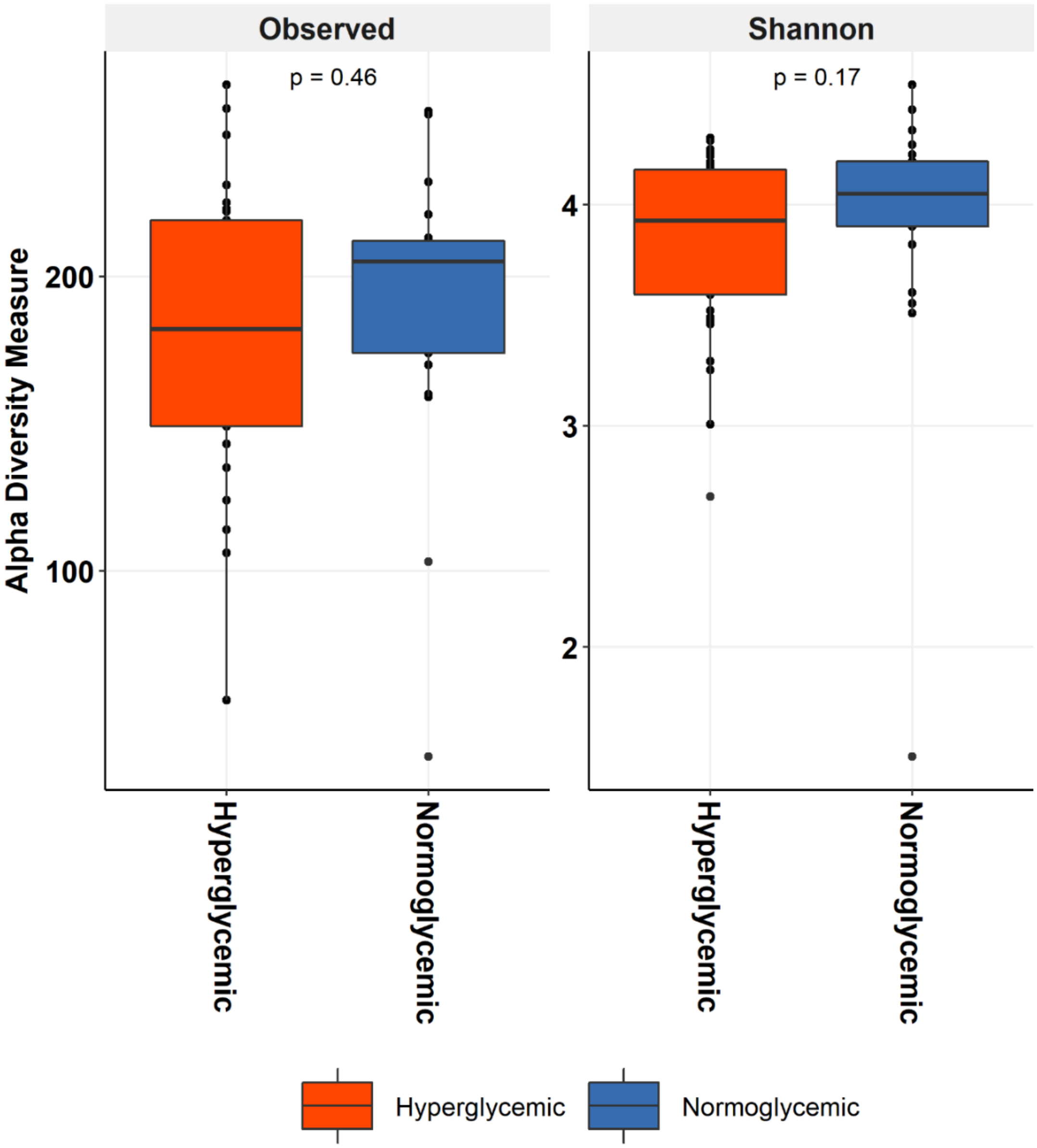

<u>Supplementary Figure 3</u>: Boxplot showing the comparison of alpha diversity (Observed richness and Shannon Diversity) between hyperglycemics and normoglycemics group in stool samples.

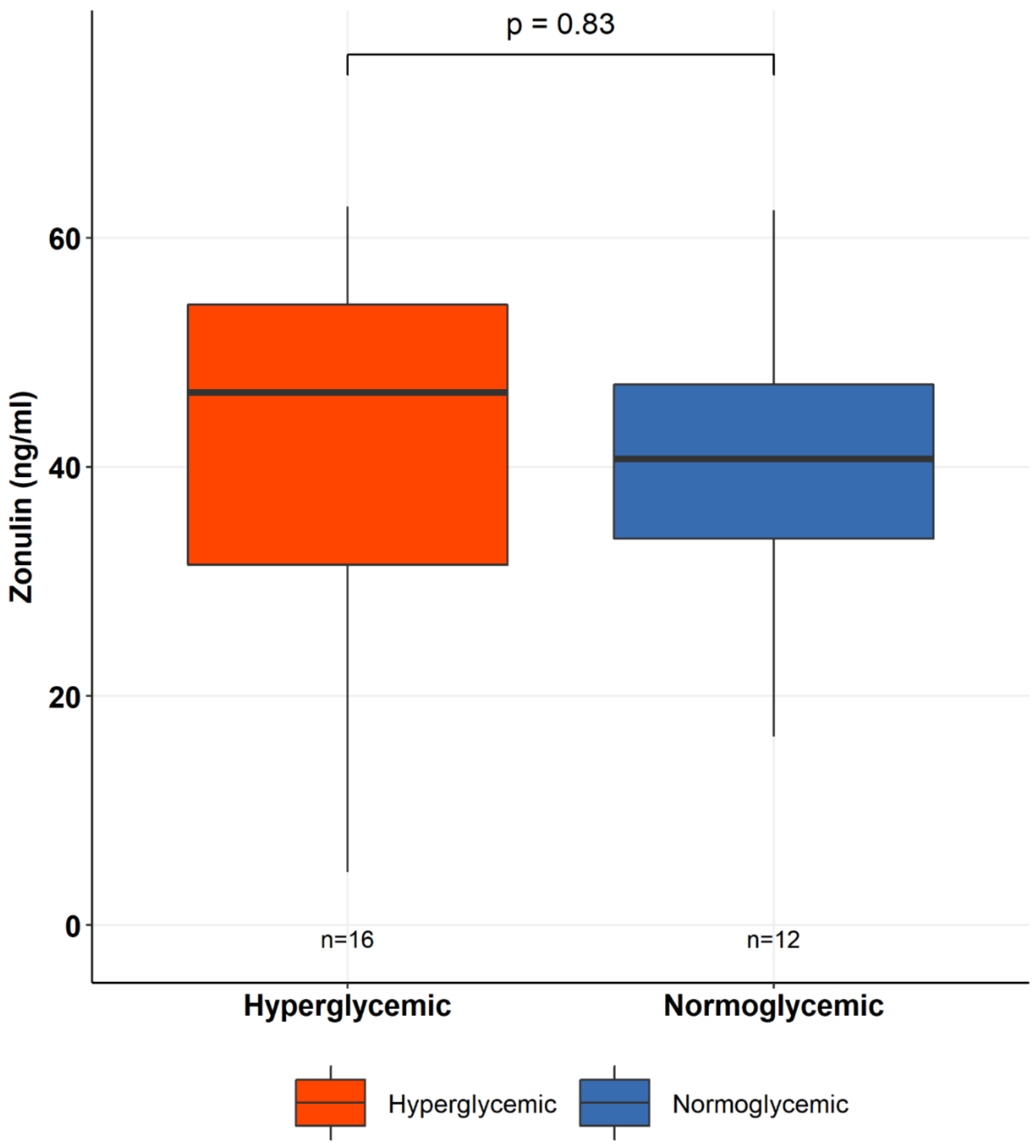

Supplementary Figure 4: Boxplot comparing the serum zonulin in hyperglycemics and normoglycemics group.

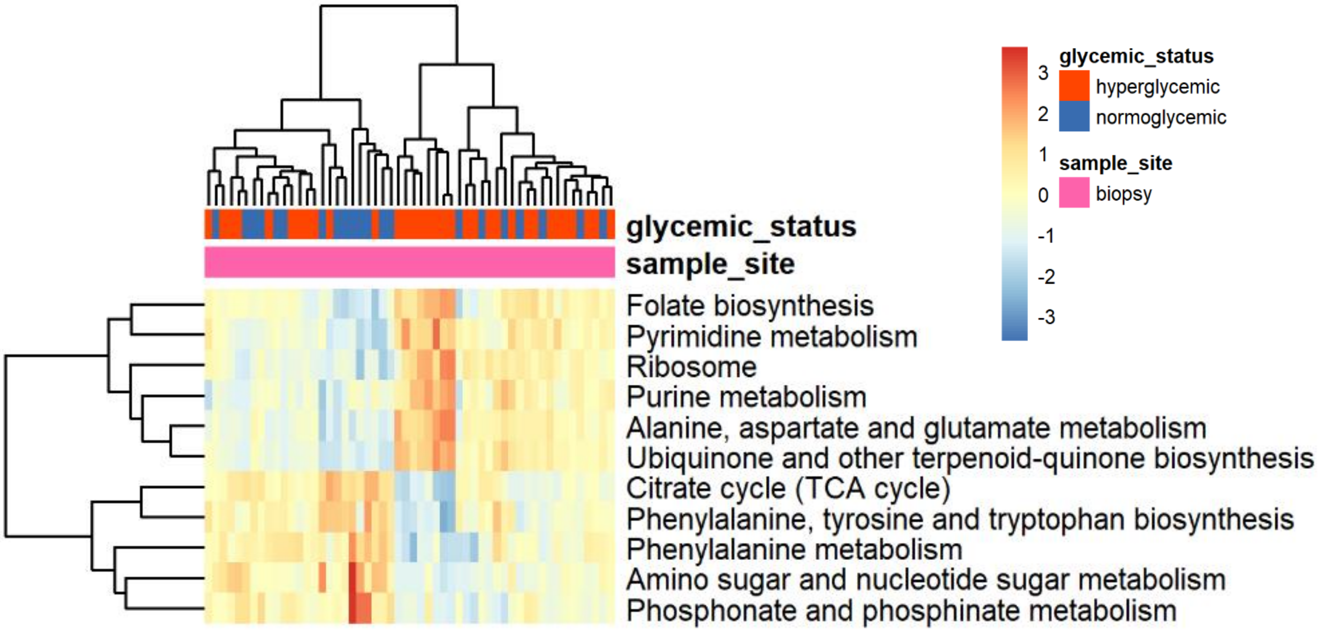

Supplementary Figure 5: Functional pathway analysis of biopsy samples. Heatmap of hierarchical clustering showing the differential significant pathways in the duodenal samples between hyperglycemic and normoglycemic. Heatmap color (blue to dark red) displays the row scaled relative abundance of each pathway across all samples.

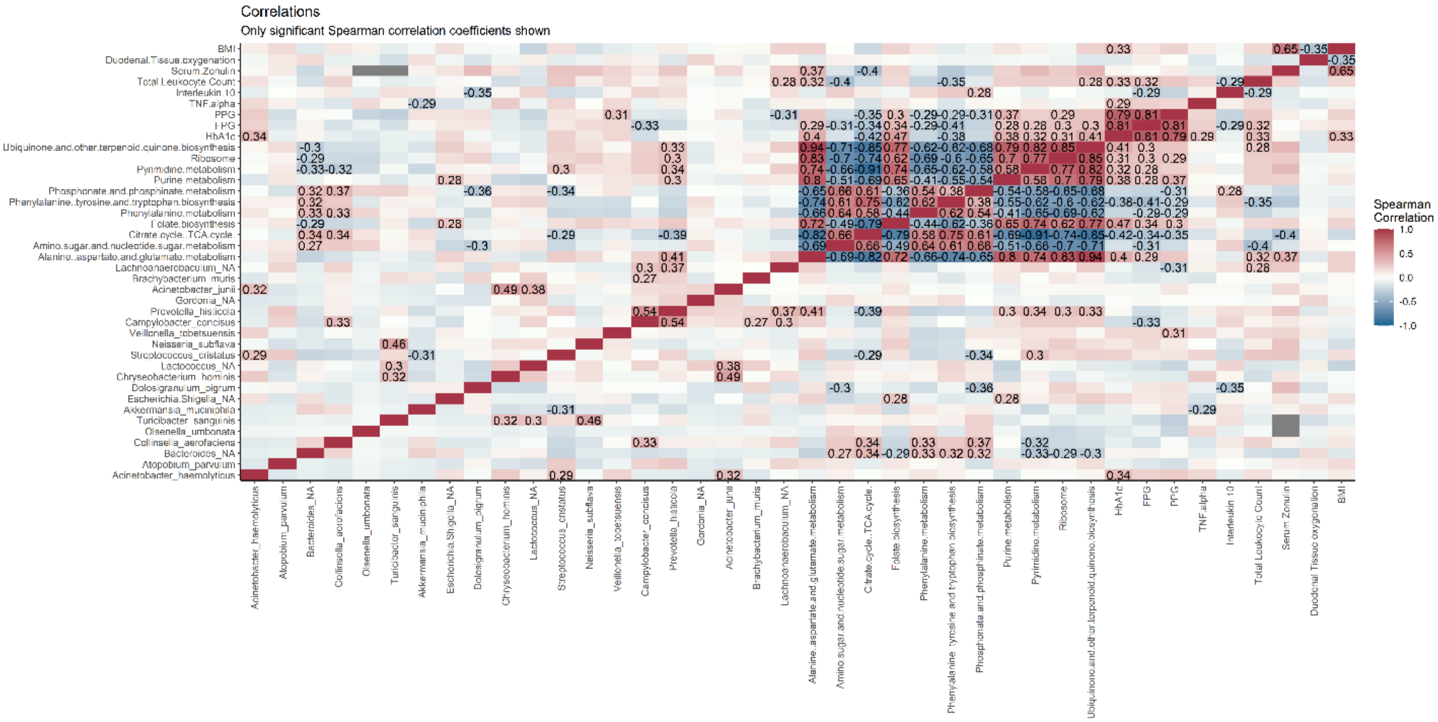

Supplementary Figure 6: **Correlation plot showing the significant spearman correlation between clinical indicators with differentially abundant species and predicted metabolic pathways. Only significant values (p<0.05) are shown.**

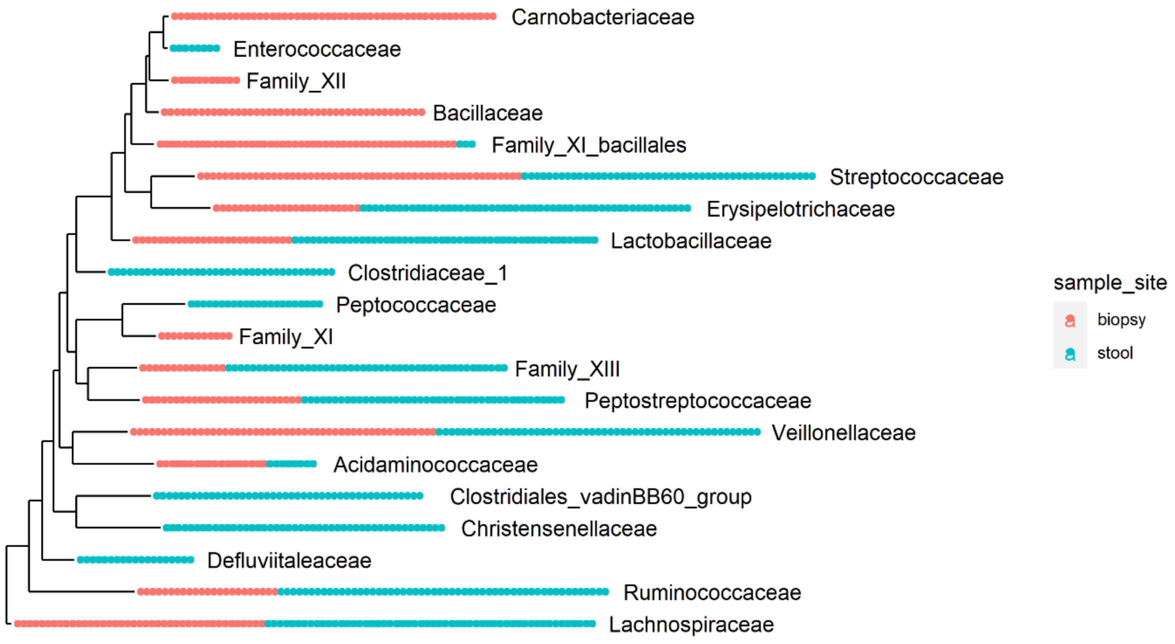

Supplementary Figure 7: **Phylogenetic tree showing the taxa that belong to Firmicutes phylum prevalent in stool and biopsy samples.**

SUPPLEMENTARY TABLES

Supplementary Table 1: Table depicting differentially abundant species in biopsy compared to stool, analyzed using DESeq2 package.

	baseMean	log2FoldChange	lfcSE	stat	pvalue	padj
Carnobacterium_maltaromaticum	1613.455734	-15.42078984	0.451980867	-34.11823588	3.96E-255	1.87E-253
Serratia_NA	1148.879907	-14.93697107	0.454171163	-32.88841804	3.22E-237	1.22E-235
Granulicatella_elegans	478.1497192	-30	0.919822352	-32.61499348	2.51E-233	7.92E-232
Stenotrophomonas_NA	275.2207093	-30	0.953713282	-31.45599474	3.48E-217	9.38E-216
Acinetobacter_NA	363.384368	-30	1.052574894	-28.50153483	1.12E-178	2.65E-177
Prevotella_nanceiensis	214.7459924	-30	1.067896861	-28.09260061	1.21E-173	2.53E-172
Sphingomonas_NA	439.8343998	-30	1.089555031	-27.53417601	6.85E-167	1.29E-165
Acinetobacter_johnsonii	535.4017573	-30	1.100107179	-27.27007021	9.61E-164	1.65E-162
Peptostreptococcus_stomatis	114.6630347	-29.85709389	1.145166477	-26.07227376	7.52E-150	1.18E-148
Porphyromonas_pasteri	324.7825	-30	1.197650456	-25.04904487	1.79E-138	2.60E-137
Gemella_NA	990.4518472	-14.65444836	0.628465258	-23.31783367	2.92E-120	3.25E-119
Bacillus_NA	254.921879	-30	1.295151784	-23.16330825	1.07E-118	1.12E-117
Prevotella_salivae	118.627363	-29.89616974	1.291260476	-23.15270257	1.37E-118	1.36E-117
Prevotella_melaninogenica	1072.714281	-14.8122896	0.65915845	-22.47151591	7.89E-112	7.45E-111
Lactobacillus_iners	206.1762066	-30	1.341449414	-22.36386977	8.85E-111	7.96E-110
Alcaligenes_faecalis	421.6215973	-30	1.389861713	-21.58488123	2.49E-103	2.05E-102
Corynebacterium_NA	102.8778318	-29.44931695	1.38082406	-21.32734922	6.33E-101	4.98E-100
Prevotella_histicola	187.8930393	-30	1.449001999	-20.70390518	3.19E-95	2.32E-94
Chryseobacterium_hominis	287.4101051	-30	1.469085312	-20.42086988	1.09E-92	7.64E-92
Phascolarctobacterium_faecium	404.7961298	-30	1.495143478	-20.06496396	1.49E-89	1.01E-88
Burkholderia_NA	160.3486125	-30	1.502215486	-19.97050375	9.94E-89	6.48E-88
Acinetobacter_bouvetii	251.868882	-30	1.546784691	-19.39507171	8.49E-84	5.35E-83
Prevotella_nigrescens	175.3361093	-29.3181995	1.537369589	-19.07036519	4.45E-81	2.71E-80
Neisseria_NA	2639.790751	-13.25825462	0.708998551	-18.69997422	4.95E-78	2.93E-77
Prevotella_NA	146.2953126	-30	1.643535769	-18.25332954	1.95E-74	1.11E-73
Gemella_morbillorum	90.09176658	-29.56606759	1.685854563	-17.53773323	7.38E-69	4.10E-68
Pseudonocardia_NA	92.51599136	-29.58122533	1.686963799	-17.5351868	7.72E-69	4.17E-68
Lachnoanaerobaculum_cf.	139.8399966	-30	1.719822193	-17.44366372	3.85E-68	1.96E-67
Prevotella_pallens	143.4741594	-30	1.802073871	-16.64748626	3.16E-62	1.49E-61
Streptococcus_NA	1288.141872	-7.856333751	0.472731033	-16.61903536	5.07E-62	2.28E-61
Fusobacterium_periodonticum	802.3212583	-13.39308519	0.80934851	-16.54798276	1.66E-61	7.11E-61
Fusobacterium_NA	60.13797789	-29.00018404	1.837130481	-15.78558754	3.91E-56	1.61E-55
Exiguobacterium_NA	93.9292709	-29.59374569	1.990087922	-14.87057198	5.12E-50	2.01E-49
Capnocytophaga_leadbetteri	37.20137672	-28.32123657	1.922715898	-14.72980828	4.15E-49	1.60E-48
Ochrobactrum_NA	87.50530846	-29.40021638	2.003761851	-14.67251029	9.67E-49	3.66E-48
Proteus_NA	102.7977377	-29.62629526	2.026283981	-14.62099861	2.06E-48	7.65E-48
Fusobacterium_nucleatum	375.0341906	-13.54910286	0.959634909	-14.11901832	2.90E-45	1.05E-44
Pseudonocardia_carboxydivorans	97.79473919	-29.69716414	2.143529984	-13.85432645	1.20E-43	4.27E-43
Providencia_NA	44.86608333	-28.59697134	2.070271795	-13.81314831	2.12E-43	7.43E-43
Flectobacillus_roseus	58.87670973	-28.86927361	2.095238131	-13.77851671	3.43E-43	1.18E-42
Pelomonas_NA	68.64555161	-29.1779978	2.168407593	-13.45595629	2.84E-41	9.42E-41
Nesterenkonia_xinjiangensis	89.32684545	-28.73295187	2.135778785	-13.45314977	2.95E-41	9.61E-41
Dialister_pneumosintes	46.35766155	-28.67180868	2.195996331	-13.05640099	5.84E-39	1.84E-38
Parvimonas_micra	25.76149898	-27.85563488	2.152176028	-12.94300955	2.57E-38	7.84E-38
Dolosigranulum_pigrum	27.93147186	-26.3956479	2.14570487	-12.30162091	8.88E-35	2.62E-34
Paracoccus_sphaerophysae	24.52192918	-27.7470726	2.28313361	-12.15306563	5.53E-34	1.61E-33
Streptococcus_cristatus	132.9406896	-8.015062225	0.6682081	-11.99485942	3.78E-33	1.08E-32
Oribacterium_parvum	32.62892972	-28.05738122	2.349879871	-11.93992151	7.33E-33	2.07E-32
Acinetobacter_haemolyticus	71.60401651	-29.02334051	2.445810645	-11.86655253	1.77E-32	4.91E-32
Stomatobaculum_longum	17.83171979	-27.33197519	2.312198458	-11.8207739	3.05E-32	8.35E-32
Leptotrichia_wadei	28.32265857	-27.72891664	2.346230959	-11.81849406	3.13E-32	8.46E-32
Rothia_NA	16.89007888	-27.27510674	2.442020039	-11.16907572	5.78E-29	1.52E-28
Rothia_mucilaginosa	1159.448647	-11.71296593	1.056327093	-11.0883892	1.43E-28	3.70E-28
Helicobacter_pylori	47.24198418	-28.63895592	2.866459959	-9.991053889	1.67E-23	4.04E-23
Curtobacterium_NA	44.27261809	-28.57125516	2.903894399	-9.838944268	7.65E-23	1.81E-22
Oribacterium_sinus	103.2842734	-13.46948536	1.371437281	-9.821437368	9.10E-23	2.12E-22
Prevotella_shahii	18.36636785	-27.09735969	2.808180291	-9.649437318	4.94E-22	1.14E-21
Acinetobacter_junii	27.30241135	-27.89110205	2.906844911	-9.594974245	8.39E-22	1.89E-21
Porphyromonas_endodontalis	16.97955892	-27.268738	2.899279565	-9.405349636	5.19E-21	1.14E-20

Rothia_NA	16.89007888	-27.27510674	2.442020039	-11.16907572	5.78E-29	1.52E-28
Rothia_mucilaginosa	1159.448647	-11.71296593	1.056327093	-11.0883892	1.43E-28	3.70E-28
Helicobacter_pylori	47.24198418	-28.63895592	2.866459959	-9.991053889	1.67E-23	4.04E-23
Curtobacterium_NA	44.27261809	-28.57125516	2.903894399	-9.838944268	7.65E-23	1.81E-22
Oribacterium_sinus	103.2842734	-13.46948536	1.371437281	-9.821437368	9.10E-23	2.12E-22
Prevotella_shahii	18.36636785	-27.09735969	2.808180291	-9.649437318	4.94E-22	1.14E-21
Acinetobacter_junii	27.30241135	-27.89110205	2.906844911	-9.594974245	8.39E-22	1.89E-21
Porphyromonas_endodontalis	16.97955892	-27.268738	2.899279565	-9.405349636	5.19E-21	1.14E-20
Rothia_dentocariosa	140.1632573	-13.85055073	1.581318622	-8.758861459	1.97E-18	4.19E-18
Gemella_sanguinis	115.7511191	-11.22117885	1.326874586	-8.456849627	2.75E-17	5.77E-17
Aggregatibacter_segnis	207.3884124	-11.38721119	1.356277688	-8.395929017	4.62E-17	9.50E-17
Haemophilus_NA	1939.86755	-4.189205334	0.532777286	-7.862957834	3.75E-15	7.54E-15
Campylobacter_NA	59.90669661	-13.67337056	1.753925607	-7.79586689	6.40E-15	1.26E-14
Lactococcus_NA	123.3397881	-13.62437548	1.755835954	-7.75948086	8.53E-15	1.66E-14
Campylobacter_concisus	134.5041413	-7.786888575	1.099671197	-7.081106242	1.43E-12	2.70E-12
Neisseria_subflava	335.7335687	-11.89549808	1.887943513	-6.300770121	2.96E-10	5.38E-10
Veillonella_atypica	824.2826995	-5.724553032	0.921302955	-6.213540295	5.18E-10	9.32E-10
Veillonella_NA	352.5651384	-5.823437399	0.938429031	-6.205517097	5.45E-10	9.72E-10
Veillonella_dispar	653.6443592	-5.196364972	0.878158074	-5.917345777	3.27E-09	5.62E-09
Veillonella_rogosae	110.424345	-7.382600896	1.500881265	-4.918844061	8.71E-07	1.44E-06
Megasphaera_micronuciformis	98.12758218	-8.803019524	1.884871609	-4.67035499	3.01E-06	4.90E-06
Veillonella_tobetsuensis	138.9414678	-8.199866944	1.756641522	-4.667922762	3.04E-06	4.91E-06
Neisseria_mucosa	106.8569018	-9.11482682	2.196094729	-4.150470696	3.32E-05	5.31E-05
Aeromonas_NA	23.49732955	-8.643822068	2.16163101	-3.99875003	6.37E-05	0.000100293
Haemophilus_parainfluenzae	346.4111515	-3.448816388	0.891279002	-3.869513789	0.000109053	0.000164888
Haemophilus_sputorum	127.7660454	-7.109530254	2.039316088	-3.486232612	0.000489875	0.000712203
Dialister_invisus	7.330159539	-7.2930326	2.170748239	-3.35968606	0.000780311	0.001117263
Eubacterium_brachy	13.64647513	-8.22577768	2.482167638	-3.313949289	0.000919882	0.001307201
Eubacterium_sulci	9.992952977	-8.040796885	2.472437996	-3.252173319	0.001145262	0.001615332
Filifactor_alocis	16.76446414	-8.7908782	2.810641688	-3.127712166	0.001761726	0.002448281
Veillonella_parvula	43.00541196	-5.768775965	1.846595722	-3.124005918	0.001784069	0.002461233
Micrococcus_lylae	16.57247425	-8.665786718	2.801357445	-3.09342413	0.001978611	0.002709837
Corynebacterium_mucifaciens	8.874001242	-7.706656296	2.899307752	-2.658102194	0.007858206	0.01053334
Gemella_parahaemolysans	9.963204523	-7.19071259	2.899787577	-2.479737705	0.013147906	0.017377302
Lautropia_mirabilis	7.723067996	-7.166182689	2.899175681	-2.471800083	0.013443467	0.017644551
Klebsiella_pneumoniae	12.06023979	-7.137240904	2.900283398	-2.460877068	0.013859785	0.017941776
Sphingobacterium_alimentarium	12.1270314	-7.144140212	2.900270707	-2.463266686	0.013767747	0.017941776
Luteimonas_tolerans	5.541083629	-6.900207768	2.900308963	-2.379128519	0.017353624	0.022311803
Gordonia_NA	5.275590278	-6.822875995	2.900429045	-2.352367835	0.018654318	0.023822068
Methylobacterium_jeotgali	6.13660632	-6.588822507	2.901207529	-2.271062115	0.023143218	0.029160455
Microbacterium_NA	1.523695446	-5.35494037	2.509655274	-2.133735428	0.032864444	0.041134967
Brachybacterium_muris	4.918158808	-6.178363207	2.901698297	-2.129223156	0.033235802	0.041326096
Actinomyces_odontolyticus	3.36722039	-5.982874887	2.893968504	-2.067360055	0.038700239	0.047806178

Supplementary Table 2: Table depicting differentially abundant species in stool compared to biopsy, analysed using DESeq2 package.

	baseMean	log2FoldChange	lfcSE	stat	pvalue	padj
Megasphaera_NA	197.5746702	27.03893545	1.127025843	23.99140678	3.42E-127	4.62E-126
Bacteroides_uniformis	70.33650864	23.13321169	0.97078811	23.8293109	1.66E-125	2.09E-124
Faecalibacterium_prausnitzii	235.1771634	9.954791384	0.421594959	23.61221637	2.89E-123	3.41E-122
Ruminococcus_bromii	35.18817687	24.77288594	1.114620267	22.22540417	1.95E-109	1.68E-108
Bifidobacterium_longum	30.50077264	24.51312347	1.161268106	21.10892682	6.59E-99	4.98E-98
Dorea_longicatena	29.5130339	6.954800201	0.396881311	17.52362734	9.46E-69	4.97E-68
Bacteroides_thetaiotaomicron	30.29770029	24.24740749	1.415270599	17.13270063	8.46E-66	4.21E-65
Alistipes_putredinis	23.84087191	24.18623168	1.43852452	16.8132217	1.95E-63	9.46E-63
Faecalibacterium_NA	108.287534	8.81950205	0.530216284	16.63378196	3.97E-62	1.83E-61
Lactobacillus_delbrueckii	9.439956684	22.95635989	1.382670896	16.6029096	6.64E-62	2.92E-61
Blautia_NA	25.89055506	6.760554335	0.409870484	16.49436736	4.03E-61	1.69E-60
Prevotella_copri	243.3688704	10.00747256	0.654546725	15.28916451	9.03E-53	3.63E-52
Blautia_massiliensis	20.93315762	6.464491017	0.473938522	13.6399358	2.32E-42	7.82E-42
Eubacterium_eligens	49.49645181	7.677029748	0.580593626	13.22272483	6.49E-40	2.08E-39
Roseburia_faecis	136.432762	9.129075081	0.701402076	13.0154663	9.99E-39	3.10E-38
Coprococcus_comes	18.48642867	6.209643127	0.503401593	12.33536646	5.84E-35	1.75E-34
Butyrivibrio_crossotus	22.65067886	23.97173654	2.070847533	11.57580949	5.47E-31	1.45E-30
Coprococcus_catus	16.33295302	5.985453853	0.557719857	10.73200779	7.20E-27	1.84E-26
Sutterella_wadsworthensis	103.1952044	8.769149082	0.82713759	10.60180216	2.92E-26	7.37E-26
Eubacterium_hallii	9.108093599	5.231137872	0.495985417	10.54695903	5.25E-26	1.30E-25
Bacteroides_NA	61.24535127	7.754113147	0.767825068	10.09880176	5.59E-24	1.37E-23
Mitsuokella_multacida	9.108265629	22.66709676	2.276442089	9.957247263	2.34E-23	5.61E-23
Fusicatenibacter_saccharivorans	10.90111414	5.403805549	0.560529176	9.640542858	5.39E-22	1.23E-21
Bacteroides_stercoris	40.84392257	24.84622467	2.637401321	9.420722009	4.48E-21	9.96E-21
Roseburia_inulinivorans	96.18501909	6.659552645	0.744733978	8.942189887	3.82E-19	8.29E-19
Odoribacter_splanchnicus	58.20607526	7.943501626	0.890955833	8.915707527	4.85E-19	1.04E-18
Mitsuokella_jalaludinii	67.8773135	7.981479732	0.950466063	8.397437891	4.56E-17	9.48E-17
Blautia_obeum	16.49929952	5.836516561	0.733517214	7.956891054	1.76E-15	3.59E-15
Ruminococcus_NA	7.217012626	4.925614034	0.627504297	7.849530368	4.18E-15	8.31E-15
Romboutsia_NA	12.89168029	5.75828144	0.749010182	7.687854685	1.50E-14	2.89E-14
Blautia_faecis	5.805876017	4.571122993	0.63127693	7.241074046	4.45E-13	8.50E-13
Slackia_isoflavoniconvertens	4.566723141	4.227291432	0.608482063	6.947273692	3.72E-12	6.97E-12
Coprococcus_eutactus	36.11758925	6.953475939	1.056723041	6.580225537	4.70E-11	8.70E-11
Roseburia_hominis	17.82179239	6.208449452	0.977595747	6.350732879	2.14E-10	3.93E-10
Bilophila_wadsworthia	23.30945385	6.60360946	1.06996704	6.171787737	6.75E-10	1.19E-09
Roseburia_intestinalis	22.96937726	6.590765895	1.090574619	6.0433883	1.51E-09	2.64E-09
Alistipes_obesi	23.24895376	6.620499325	1.109417901	5.967543265	2.41E-09	4.18E-09
Alistipes_senegalensis	19.74596863	6.350694678	1.077009217	5.89660198	3.71E-09	6.32E-09
Anaerostipes_hadrus	5.230760655	4.458208804	0.762657981	5.845620072	5.05E-09	8.52E-09
Holdemanella_biformis	15.17594023	5.968628946	1.202433381	4.963791792	6.91E-07	1.16E-06
Lactobacillus_ruminis	115.8026381	4.527815631	0.922440517	4.90851773	9.18E-07	1.51E-06
Ruminococcus_callidus	5.998896335	4.664045909	1.134493162	4.111127385	3.94E-05	6.25E-05
Dorea_formicigenerans	10.17736589	2.348375489	0.595885723	3.940982977	8.11E-05	0.000126752
Flavonifractor_plautii	5.292811925	4.472615289	1.140117985	3.922940739	8.75E-05	0.000135514
Clostridium_NA	3.832864782	4.021133524	1.026617405	3.916876437	8.97E-05	0.000137837
Ruminococcus_bicirculans	8.034208248	5.084247005	1.300836615	3.908443956	9.29E-05	0.000141586
Lachnospiraceae_bacterium	4.589092292	4.155891908	1.080310514	3.846942017	0.000119601	0.000179402
Allisonella_histaminiformans	6.833264595	4.681266652	1.235301777	3.789573318	0.000150906	0.000224577
Parasutterella_excrementihominis	6.322664811	4.548133825	1.24152455	3.663345862	0.000248942	0.000367578
Bifidobacterium_NA	211.9075037	2.635239217	0.722158391	3.649115276	0.000263145	0.000385538
Parabacteroides_distasonis	22.57697765	2.950428975	0.876472179	3.366255135	0.000761962	0.001099319
Intestinibacter_bartlettii	3.035093413	3.654111323	1.124689883	3.24899457	0.001158137	0.001621392
Escherichia/Shigella_NA	280.3165491	2.433914347	0.802090093	3.034465042	0.002409627	0.003276399
Alistipes_shahii	5.754964555	4.411673322	1.608029704	2.743527257	0.0060783	0.008205705
Klebsiella_NA	20.15517261	3.951550477	1.504537497	2.626422063	0.008628775	0.011484778
Bifidobacterium_angulatum	5.417323415	4.460479096	1.918217259	2.325325286	0.020054573	0.025438351

<u>Supplementary Table 3</u>: Table depicting the Spearman correlations of zonulin with anthropometric variables and bacterial load.

Variable	Correlation Coefficient	95% CI	p value
**Height (m)**	-0.46	1.59, 1.66	0.012
**Weight (Kg)**	0.48	69.5, 78.6	0.009
**Body Mass Index (Kg/m2)**	0.65	26.0, 30.8	0.0001
**Waist Circumference (in.)**	0.47	38.0, 41.7	0.009
**Hip Circumference (in.)**	0.53	39.2, 42.3	0.003
**Total Bacterial Count (/ng of DNA)**	0.36	474.1, 2354.4	0.061<br>(#0.03, one-sided)
